# RNA Granules Hitchhike on Lysosomes for Long-Distance Transport, Using Annexin A11 as a Molecular Tether

**DOI:** 10.1016/j.cell.2019.08.050

**Published:** 2019-09-19

**Authors:** Ya-Cheng Liao, Michael S. Fernandopulle, Guozhen Wang, Heejun Choi, Ling Hao, Catherine M. Drerup, Rajan Patel, Seema Qamar, Jonathon Nixon-Abell, Yi Shen, William Meadows, Michele Vendruscolo, Tuomas P.J. Knowles, Matthew Nelson, Magdalena A. Czekalska, Greta Musteikyte, Mariam A. Gachechiladze, Christina A. Stephens, H. Amalia Pasolli, Lucy R. Forrest, Peter St George-Hyslop, Jennifer Lippincott-Schwartz, Michael E. Ward

**Affiliations:** 1 HHMI Janelia Research Campus, Ashburn, VA, USA; 2 NINDS, NIH, Bethesda, MD, USA; 3 Cambridge Institute for Medical Research, Department of Clinical Neurosciences, University of Cambridge, Cambridge CB2 0XY, UK; 4 NICHD, NIH, Bethesda, MD, USA; 5 Department of Chemistry, University of Cambridge, Cambridge CB2 1EW, UK; 6 Cavendish Laboratory, Department of Physics, University of Cambridge, Cambridge CB3 0HE, UK; 7 Department of Medicine (Division of Neurology), University of Toronto and University Health Network, Toronto, Ontario M5S 3H2, Canada

**Keywords:** RNA transport, Lysosome, RNA granule, Phase separation, Neuron, Local translation, ANXA11, Amyotrophic lateral sclerosis, Neurodegeneration, Organelles contact

## Abstract

Long-distance RNA transport enables local protein synthesis at metabolically-active sites distant from the nucleus. This process ensures an appropriate spatial organization of proteins, vital to polarized cells such as neurons. Here, we present a mechanism for RNA transport in which RNA granules “hitchhike” on moving lysosomes. *In vitro* biophysical modeling, live-cell microscopy, and unbiased proximity labeling proteomics reveal that annexin A11 (ANXA11), an RNA granule-associated phosphoinositide-binding protein, acts as a molecular tether between RNA granules and lysosomes. ANXA11 possesses an N-terminal low complexity domain, facilitating its phase separation into membraneless RNA granules, and a C-terminal membrane binding domain, enabling interactions with lysosomes. RNA granule transport requires ANXA11, and amyotrophic lateral sclerosis (ALS)-associated mutations in ANXA11 impair RNA granule transport by disrupting their interactions with lysosomes. Thus, ANXA11 mediates neuronal RNA transport by tethering RNA granules to actively-transported lysosomes, performing a critical cellular function that is disrupted in ALS.

## Highlights

• RNA granules “hitchhike” on motile lysosomes during long-distance transport
• ANXA11 binds to RNA and lysosomes via phase separating and membrane binding domains
• ANXA11 tethers RNA granules to lysosomes and is required for axonal RNA transport
• ALS-associated ANXA11 mutations impair its tethering function and RNA transport

## Graphical Abstract

**Figure:**
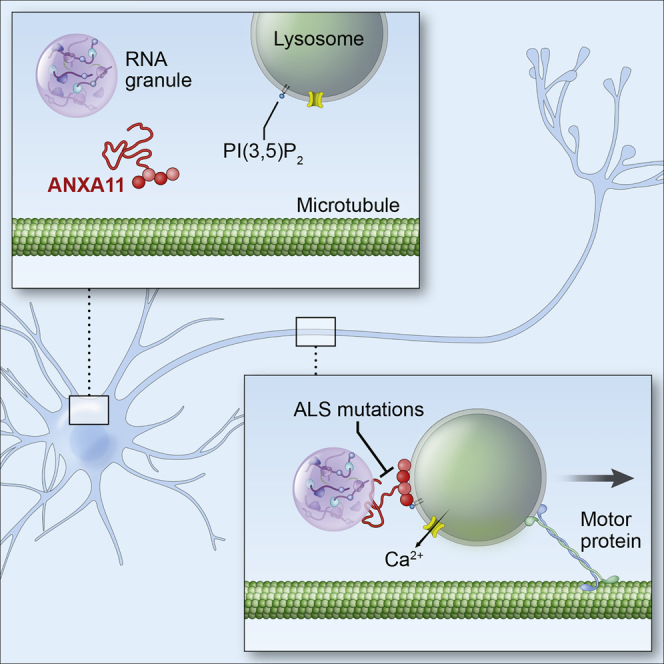

## Teaser

Annexin A11, a protein with mutations associated with ALS, tethers membraneless RNA granules to actively-transported lysosomes via its intrinsic membrane-binding and phase separating properties, enabling efficient transport of RNA to distal regions of the neuron.

## Introduction

Many proteins within cells are translated locally rather than trafficked from their site of synthesis to their final destination. Neurons, which have long axons and dendrites, rely on local translation for numerous cell-specific functions (Glock et al., 2017, Jung et al., 2012, Krichevsky and Kosik, 2001, Leung et al., 2006, Martin and Ephrussi, 2009, Wong et al., 2017, Yao et al., 2006, Zheng et al., 2001). Local translation requires long-distance transport of RNA from the nucleus to distal parts of the cell.

For membrane-bound organelles such as mitochondria and endosomes, the microtubule-based motors kinesin and dynein interact either directly or indirectly with membrane proteins and lipids to enable long-range transport. RNAs, however, do not usually exist in membrane-enclosed structures. Instead, they interact with with RNA-binding proteins (RBPs), which self-organize into phase separated structures called RNA granules (Weber and Brangwynne, 2012). RNA granules have long been observed to traffic within neuronal axons and dendrites (Knowles et al., 1996, Gopal et al., 2017). While their transport requires both microtubules and motor proteins, how membraneless RNA granules are tethered to transport machinery remains incompletely understood (Clark et al., 2007, Davidovic et al., 2007, Dictenberg et al., 2008, Dienstbier et al., 2009, Dix et al., 2013, Gáspár et al., 2017, Gagnon et al., 2013).

Mutations in RBPs, molecular motors, and microtubule components have all been linked to neurological diseases, highlighting the critical contributions of RNA transport and metabolism to long-term neuronal integrity (Baird and Bennett, 2013, Bakthavachalu et al., 2018, Chevalier-Larsen and Holzbaur, 2006, Fallini et al., 2011, Hirokawa et al., 2010, Puls et al., 2003, Ramaswami et al., 2013). In particular, numerous causative mutations for amyotrophic lateral sclerosis and frontotemporal dementia (ALS/FTD), two related adult-onset neurodegenerative diseases, fall within these gene groups (Zhang et al., 2015, Murakami et al., 2015, Van Deerlin et al., 2008, Kim et al., 2013, Münch et al., 2005, Nicolas et al., 2018, Vance et al., 2009). The bulk of other genes linked to familial ALS/FTD encode proteins that regulate lysosomal biology (Baker et al., 2006, Guerreiro et al., 2015, Pottier et al., 2015, Renton et al., 2011, Shi et al., 2018, Skibinski et al., 2005, Ward et al., 2017). Lysosomes and lysosome-related organelles are coupled to motor proteins through well-characterized adapter proteins, and like RNA granules, lysosomes traffic long distances within neuronal processes (Farías et al., 2017, Fu and Holzbaur, 2014, Pu et al., 2015).

Recently, it has become clear that not all cargos directly interact with motor proteins during long-range transport. Rather, some cargos are indirectly transported along microtubule networks by docking onto other membrane-bound organelles such as endosomes, a process known as “hitchhiking” (Guimaraes et al., 2015, Salogiannis et al., 2016, Salogiannis and Reck-Peterson, 2017). Endosomal hitchhiking appears to be the primary mechanism by which peroxisomes, lipid droplets, and ER travel long-distances within filamentous fungi. Interestingly, RNA granules also hitchhike on moving endosomes in filamentous fungi during long-distance trafficking, hinting at the possibility of similar phenomena in higher-order organisms (Baumann et al., 2012, Higuchi et al., 2014, Pohlmann et al., 2015).

In this study, we show that RNA granules hitchhike on lysosomes for long-distance trafficking in mammalian cells. Using a combination of proximity labeling proteomics, live-cell imaging, and *in vitro* assays, we then identify the ALS-associated protein ANXA11 as a molecular tether that can dynamically couple RNA granules with lysosomes. ALS-associated mutations in ANXA11 disrupt docking between RNA granules and lysosomes, consequently impeding RNA granule transport in neurons *in vitro* and *in vivo*. Together, these findings identify the lysosome as a key player in neuronal RNA transport, characterize how ANXA11 enables interactions between membraneless RNA granules and lysosomes, and provide mechanistic evidence for the involvement of altered RNA transport in ALS pathogenesis.

## Results

### RNA Granules Hitchhike on Motile Lysosomes in Mammalian Cells

**Figure 1:**
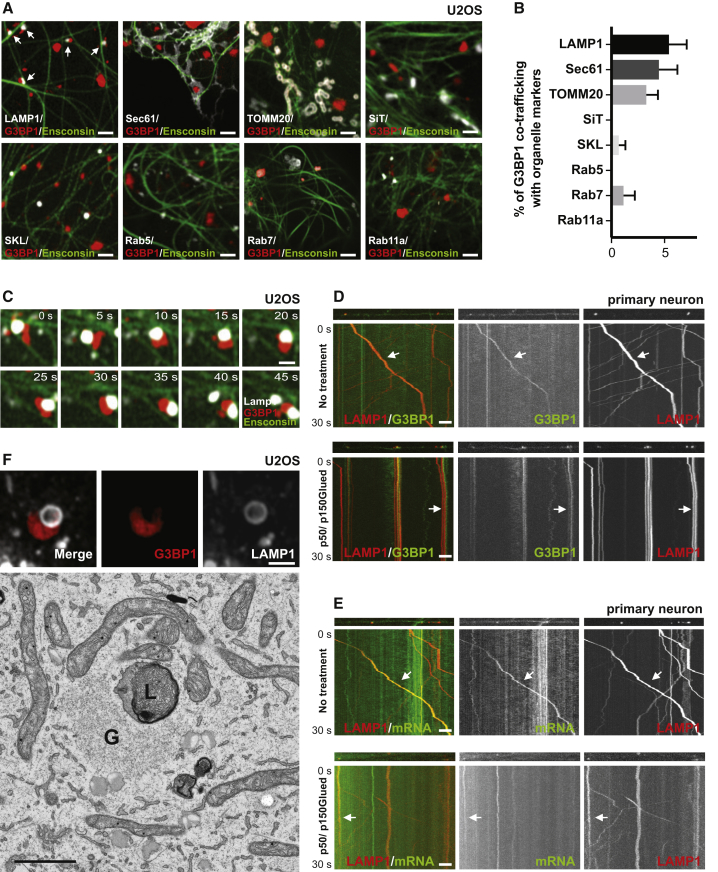
RNA Granules Hitchhike on Motile Lysosomes in Mammalian Cells (A) RNA granule co-imaging with different organelles. U2OS cells expressing mCherry-G3BP1 and different organelle markers were imaged live 30 minutes after heat shock (43^o^C). Organelle markers: LAMP1 – lysosome, Sec61 – ER, TOMM20 – mitochondria, SiT – Golgi, SKL – peroxisome, Rab5 – early endosome, Rab7 – late endosome, Rab11a – recycling endosome, Ensconsin – microtubule. Arrows point to lysosome-RNA granule contact sites. Scale bar: 2μm. See also Figures S1A, S1B. (B) Percentage of RNA granules that co-traffic with different organelles from (*A*). N=7. (C) Time-lapse image sequence showing RNA granule (mCherry-G3BP1) co-trafficking with a lysosome (LAMP1-HaloTag) along a microtubule (Ensconsin-GFP) in U20S cells immediately after heat shock at 43^o^C. Scale bar: 1μm. See also Figure S1F, Videos S1, S2. (D) Kymograph of RNA granules co-trafficking with lysosomes in axons. Axons of rat cortical neurons expressing LAMP1-HaloTag and mEmerald-G3BP1 were imaged at 100ms/frame for 30 seconds. Arrow points to a lysosome co-trafficking with a G3BP1-labeled structure. P50/p150Glued: Doxycycline-inducible expression of a p50 dynactin subunit and the CC1 domain of the p150 glued subunit of dynactin was used to inhibit motor-directed transport of lysosomes. Scale bar: 5 μm. See also Figures S1J, S1K. (E) Kymograph of mRNA co-trafficking with lysosomes in axons. Axons of rat cortical neurons expressing LAMP1-HaloTag, actin-24xMBS and MCP-NLS-2xEGFP were imaged as in (D). Arrow points to a lysosome co-trafficking with actin mRNA. Scale bar: 5 μm. See also Figure S1I. (F) CLEM images of an RNA granule associated with a lysosome. Upper panel shows the fluorescent image of a LAMP1-labeled lysosome and a G3BP1-labeled RNA granule. Lower panel shows the correlated electron microscopy image. L, lysosome; G, RNA granule. Scale bar: 1μm.

**Figure S1:**
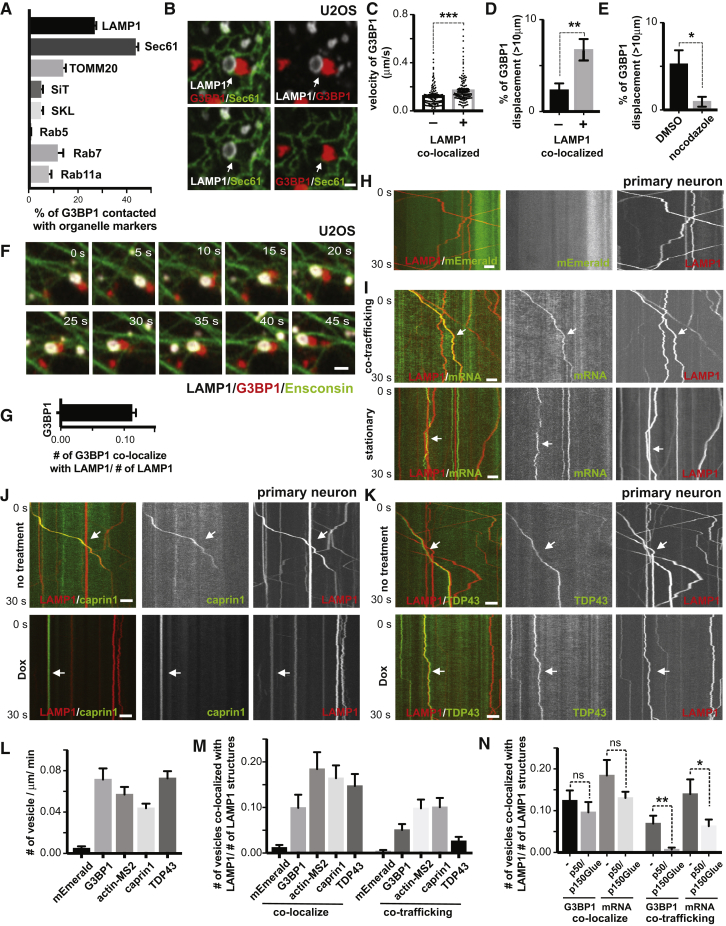
RNA Granules Hitchhike on Motile Lysosomes in Mammalian Cells, Related to Figure 1 (A) Quantification of the percentage of RNA granules in contact with different organelles from Figure 1A. (n=7). (B) Contacting RNA granules and lysosomes are frequently in close association with ER. U20S cells were transfected with LAMP1-HaloTag, mEmerald-SEC61 and low levels of mCherry-G3BP1 for 24hrs. Cells were imaged live for 30 minutes after heat shock (43^o^C). Arrows point to areas where co-localized LAMP1 (white)- and G3BP1 (red)- labeled structures are in close association with Sec61-labeled ER (green). Scale bar: 1μm. (C) Quantification of velocity of G3BP1 labeled RNA granule co-localized or not co-localized with lysosomes, n= 455 (number of granules, not co-localized), 396 (number of granules, co-localized), t-test, ^∗∗∗^p < 0.001. Error bars = SEM. (D) Percentage of G3BP1 labeled RNA granule co-localized or not co-localized with lysosomes with displacement over 10μm, n=7 (number of cells), t-test, ^∗∗^p < 0.01. Error bars = SEM. (E) Percentage of G3BP1 labeled RNA granules treated or not treated with nocodazole (Nadezhdina et al. 2010) with displacement over 10μm, n=4, t-test, ^∗^p < 0.05. Error bars = SEM. (F) Time-lapse image sequence showing an RNA granule co-trafficking with a lysosome along a microtubule. U2OS cells were transfected with LAMP1-HaloTag, Ensconsin-GFP and low amounts of mCherry-G3BP1 for 24hrs. Images were acquired immediately after heat shock at 43^o^C. Scale bar: 1μm. (G) Quantification of LAMP1 labeled lysosomes co-localizing with G3BP1 labeled RNA granules (relative to number of lysosome), n=20 (number of cells). (H) Kymograph of mEmerald tag and lysosomes in axons. Rat cortical neurons were transduced with LAMP1-HaloTag to label lysosomes and PGK promoter driven mEmerald tag. Time-lapse images of axons were acquired at 100ms/frame for 30 seconds. Scale bar: 5 μm. (I) Kymographs illustrating co-trafficking and stationary interaction patterns of lysosomes with RNA granules. Rat cortical neurons were transduced with LAMP1-HaloTag to label lysosomes and actin-24xMBS/MCP-NLS-2xEGFP to label actin mRNA. Upper panel shows co-trafficking of lysosomes and mRNA, and bottom panel shows lysosomes and mRNA associating in a relatively stationary manner. Scale bar: 5 μm. (J) Kymograph of CAPRIN1-labeled RNA granules co-trafficking with lysosomes in axons. Rat cortical neurons were transduced with LAMP1-HaloTag to label lysosomes and mEmerald-CAPRIN1 to label RNA granules. Time-lapse images of axons were acquired at 100ms/frame for 30 seconds. Arrows point to lysosomes co-trafficking with CAPRIN1-labeled structures. p50/p150Glued, doxycycline-inducible expression of a p50 dynactin subunit and the CC1 domain of the p150 glued subunit of dynactin. Scale bar: 5 μm. (K) Kymograph of TDP43-labeled RNA granules co-trafficking with lysosomes in axons. Rat cortical neurons were transduced with LAMP1-HaloTag to label lysosomes and mEmerald-TDP43 to label RNA granules. Time-lapse images of axons were acquired at 100ms/frame for 30 seconds. Arrow points to a lysosome co-trafficking with a TDP43-labeled structure. Dox, doxycycline-inducible expression of a p50 dynactin subunit and the CC1 domain of the p150 glued subunit of dynactin. Scale bar: 5 μm. (L) Quantification of frequency of G3BP1, actin-MS2, CAPRIN1, TDP43 labeled RNA granule and mEmerald tag in axons, n=22(mEmerald), 19(G3BP1), 35(actin-MS2), 21(caprin1), 35(TDP43). (M) Quantification of LAMP1 labeled lysosomes co-localizing or co-trafficking with G3BP1, actin-MS2, CAPRIN1, TDP43 labeled RNA granules and mEmerald tag (relative to number of lysosomes) in axons, n=22(mEmerald), 19(G3BP1), 36(actin-MS2), 25(CAPRIN1), 41(TDP43). (N) Quantification of LAMP1-labeled lysosomes co-localizing or co-trafficking with G3BP1, actin-MS2 (relative to number of lysosomes), with or without doxycycline-inducible expression of a p50 dynactin subunit and the CC1 domain of the p150 glued subunit of dynactin. N=35(G3BP1,-), 35(G3BP1, p50/p150Glued), 36(actin-MS2, -), 30(actin-MS2, p50/p150Glued). T-test, ^∗∗^p < 0.01, ^∗^p < 0.05, ns, not significant. Error bars = SEM.

Using live-cell microscopy, we explored whether RNA granules could move within mammalian cells through association with motile, membranous organelles. Following heat shock, G3BP1-labeled RNA granules predominately co-localized with markers for lysosomes (LAMP1) and ER (SEC61) in U2OS cells (Figure1A, Figures S1A and S1B) and co-trafficked with lysosomes (Figure 1B, Figures S1C and S1D, Video S1). Lysosomes and juxta-positioned RNA granules moved along microtubules, and their motility was blocked by nocodazole-induced microtubule depolymerization (Figure 1C and Figures S1E and S1F). Thus, stress-induced RNA granules co-traffic with lysosomes during microtubule-dependent transport.

Video S1. RNA granule/lysosome co-trafficking in U2OS cells. Related to Figure 1

Video S2. mRNA co-trafficking with lysosomes in U2OS cells. Related to Figure 1

Next, we analyzed RNA granule and LAMP1 dynamics in cultured primary cortical neurons, which constitutively transport these structures within axons. In neurons, LAMP1-positive vesicles include both degradative lysosomes and non-acidic endo-lysosomes (Cheng et al., 2018, Farías et al., 2017), but for simplicity, we hereafter refer to all LAMP1 positive structures as lysosomes. Within axons, lysosomes co-trafficked with RNA granules labeled with G3BP1 (Sahoo et al. 2018), TDP-43 (Alami et al., 2014, Gopal et al., 2017), and CAPRIN1 (Nakayama et al., 2017) (Figure 1D, Figures S1J and S1K). Lysosomes also co-trafficked with actin-MS2/MCP (Figure 1E, Figure S1I), a probe that labels actin mRNA, one of the most abundant mRNAs in axons (Bassell et al., 1998). Of note, although most anterograde and retrograde moving RNA granules clearly co-trafficked with lysosomes (Figures 1D and 1E), only a fraction of lysosomes co-trafficked with RNA granules (Figures S1H, S1L, and S1M). Inhibition of motor-dependent lysosomal movement blocked RNA granule transport (Figures 1D and 1E, Figure S1N). These results confirm that trafficking of RNA granules and lysosomes during long-range axonal transport are intimately linked.

In confocal images, RNA granules and lysosomes were closely apposed (Figure 1F, top panel). Correlative light-electron microscopy (CLEM) imaging of these structures confirmed that lysosomes and RNA granules were tightly associated, with no evidence of lysosomal engulfment of the RNA granule (as would be expected during autophagy) (Buchan et al., 2013) (Figure 1F, bottom image). These results support a model in which lysosomes serve as a vehicle for RNA granule transport, and suggest the presence of a molecular tether between RNA granules and lysosomes.

### Identification of ANXA11 as a Mediator of RNA Granule-Lysosome Associations

**Figure 2:**
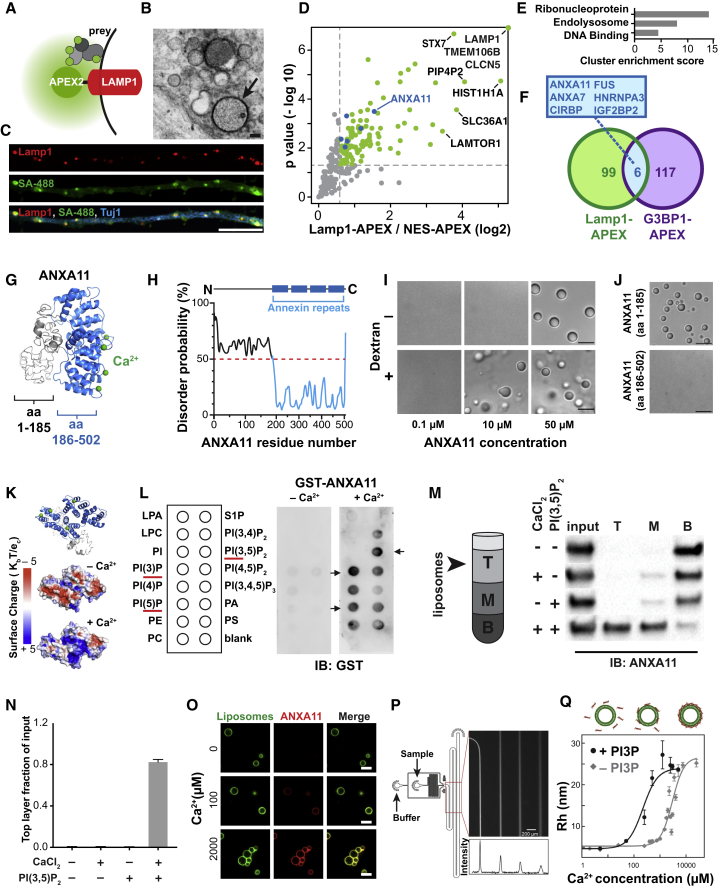
Identification of ANXA11 as a Potential Mediator of RNA Granule-Lysosome Associations (A–G) Proximity labeling proteomic screen for lysosomal interacting proteins in i^3^Neurons. (A) Schematic of LAMP1-APEX2 bait. (B) Electron microscopy image of DAB precipitate generated by LAMP1-APEX2 (dark contrast, arrow) surrounding lysosomes in i^3^Neurons. Scale bar: 100 nm. (C) Confocal immunofluorescence image of LAMP1-APEX2 biotinylated prey (streptavidin-488 staining) surrounding LAMP1-positive lysosomes in i^3^Neuron axons (Tuj1). Scale bar: 10 μm. (D) Plot showing statistically significant LAMP1-APEX2 enriched prey proteins from proximity-labeling proteomics in i^3^Neurons. n = 4, p values corrected for multiple comparisons. (E) Functional Annotation Clustering of DAVID Gene Ontology terms of Lamp1-APEX enriched prey. (F) Venn diagram of LAMP1-APEX2 hits versus G3BP1-APEX2 stress-granule hits (Markmiller et al. 2018). Overlapping hits are also represented as blue dots in (D). (G) Predicted structural analysis of ANXA11 revealed four C-terminal calcium-binding annexin repeats (blue), and a disordered N-terminal region. (H–J) Recombinant ANXA11 undergoes liquid-liquid phase separation *in vitro.* (H) PrDOS analysis of ANXA11 predicted a high likelihood of disorder of aa 1-185. (I) Full-length ANXA11 formed spherical, fusing liquid droplets at concentrations above 50μM (upper panel). Phase separation of ANXA11 was facilitated by 10% dextran, with phase separation occurring at lower ANXA11 concentrations (≥ 10μM). Scale bar: 5 μm. See also Figure S2A. (J) The disordered N-terminus (aa 1-185, upper panel) of ANXA11 but not C-terminus (aa 186-502, lower panel) underwent liquid-liquid phase separation. Scale bar: 5 μm. (K–Q) Recombinant ANXA11 interacts with negatively charged lysosome-associated phospholipids. (K) Surface maps of predicted ANXA11 structure +/– Ca^2+^ showing increased positive surface charge (blue) in the presence of Ca^2+^. Top panel shows orientation of ANXA11 and location of Ca^2+^ ions (green). (L) Protein lipid overlay assay of recombinant ANXA11-GST protein with membrane lipids. Recombinant ANXA11-GST protein was incubated with a membrane lipid strip +/– Ca^2+^, followed by anti-GST immunoblotting. Arrowheads indicate enriched lipid binding. Red line highlighted the correlated phospholipid species. (M) Liposome flotation assay of recombinant ANXA11 with liposomes containing PI(3,5)P2 in the absence or presence of Ca^2+^. Liposomes with associated proteins floated to the top layer following ultra-centrifugation (schematic). ANXA11 in the top (T), middle (M) and bottom (B) fractions was detected via anti-ANXA11 western blot. (N) Quantification ANXA11 enrichment in the top liposome fraction in Figure 2M. n = 3, error bars = SEM. (O) Microscopy analysis of calcium-dependent recruitment of recombinant ANXA11 protein to fluorescent PI3P-containing liposomes. Representative images showed ANXA11 binding to PI3P-containing liposomes at the indicated calcium concentrations. Scale bar, 5μm. (P) Microfluidic device design for diffusional sizing assay of calcium-dependent ANXA11 binding to liposomes. Inset indicates detection area. The fluorescence intensity along the channel indicates different diffusion times of ANXA11. Scale bar, 200 μm. (Q) Microfluidic diffusional sizing assay to assess changes in molecular radius of ANXA11 upon Ca^2+^-dependent binding to liposomes (top panel). Bottom panel: Quantification of hydrodynamic radius of ANXA11 when binding to liposomes with (dots) and without (diamonds) PI3P versus Ca^2+^ concentration. Data were fitted with a Hill binding model.

To identify potential proteins that tether RNA granules to lysosomes, we used ascorbate peroxidase (APEX) proximity labeling proteomics (Hung et al., 2016) to label and catalog the interactome of LAMP1-positive lysosomes (LAMP1-APEX2 probe) in human iPSC-derived neurons (i^3^Neurons) (Fernandopulle et al., 2018) (Figures 2A and 2B). Immunofluorescence staining of biotinylated prey confirmed that we could successfully label proximal lysosome-interacting proteins in i^3^Neurons and axonal compartments (Figure 2C). We identified ∼130 proteins as LAMP1-APEX2 prey by proteomic mass spectrometry in i^3^Neurons (using nuclear export signal APEX [NES-APEX2] as a spatial reference), representing proteins that are specifically associated with lysosomes (Figure 2D). Gene Ontology-term analysis of these prey showed substantial labeling of lysosomal proteins, as expected, along with a significant enrichment of RNA granule-associated proteins (Figure 2E). To identify proteins that might function as a molecular tether between lysosomes and RNA granules, we cross-referenced our list with a previously generated list of RNA granule interacting proteins that used G3BP1-APEX2 as a bait (Markmiller et al., 2018) (Figure 2F). Through this analysis, we identified six putative interacting partners of both lysosomes and RNA granules. Of these, annexin A11 (ANXA11) was the highest-ranked lysosome-interacting protein based on LAMP1-APEX2 proteomics.

Mutations in ANXA11 are associated with ALS, a neurodegenerative disease in which dysfunction of lysosomal and RNA granule biology play causal roles (Smith et al., 2017, Tsai et al., 2018, Zhang et al., 2018a). We performed structural modeling of ANXA11 as a first step in its characterization (Figure 2G). ANXA11, like other annexin family members, contains four C-terminal calcium-dependent membrane-binding annexin domains. Unlike most other annexin family members, however, ANXA11 also has a long N-terminal low-complexity region (LCR) (Figure 2H). LCRs are common to RNA granule-associated proteins, and facilitate formation of transient phase-separated assemblies that shelter associated RNAs (Hyman and Brangwynne, 2011, Hyman et al., 2014, Weber and Brangwynne, 2012). Thus, ANXA11 contains structural features that could enable both binding to lipid membranes (e.g., lysosomes) and intercalation within phase-separated RNA granules.

**Figure S2:**
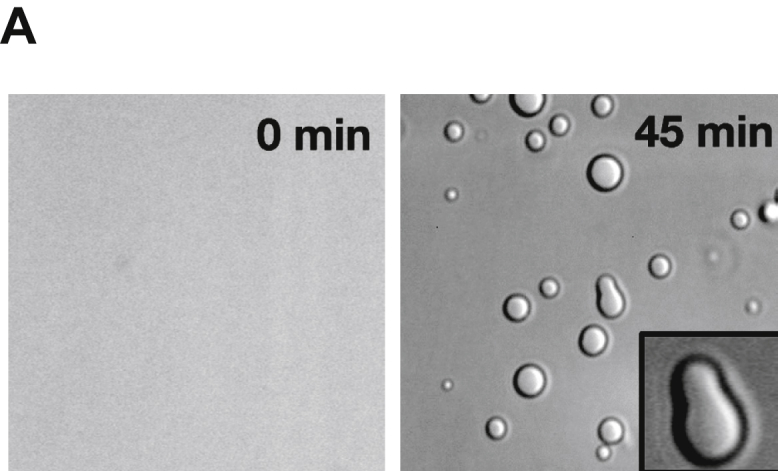
Recombinant ANXA11 Undergoes Liquid-Liquid Phase Separation *In Vitro,* Related to Figure 2 A. Purified ANXA11 protein formed biological condensates. Full-length wild type ANXA11 formed spherical, fusing liquid droplets at ANXA11 concentrations at 10μM facilitated by 10% dextran. Inset shows a fusion event between two phase separated liquid droplets.

We used a series of *in vitro* assays to characterize the biophysical properties of ANXA11. At high concentrations, or when incubated with 10% dextran (a molecular crowding agent), purified ANXA11 formed phase-separated droplets that grew in size and fused with each other over time (Figure 2I, Figure S2A). A similar change occurred when ANXA11 was transitioned from 4^o^C to 25^o^C. We performed the same assays with purified ANXA11 N terminus (amino acids 1-185; the LC region) and ANXA11 C terminus (amino acids 186-502; the annexin region). As predicted by our structural models, the N-terminal LCR region of ANXA11 was necessary and sufficient for phase separation (Figure 2J). These results indicate that ANXA11 can form phase-separated droplets similar to traditional RNA granule proteins, and that the N terminus of ANXA11 confers this property.

We next investigated whether purified ANXA11 could bind membrane lipids. Structural modeling predicted that calcium binding conferred a positive surface charge to ANXA11’s annexin domains (Figure 2K), which could potentiate binding of ANXA11 to negatively-charged, membrane phospholipids. Using a protein lipid overlay assay, we found that ANXA11 bound several lysosome-enriched, negatively-charged phosphatidylinositols in a Ca^2+^-dependent manner (Figure 2L). Three-dimensional lipid flotation lipid overlay assays confirmed that ANXA11 co-floated with PI(3,5)P_2_ containing liposomes (Figures 2M and 2N) and interacted with PI3P-containing liposomes in a Ca^2+^-dependent manner (Figure 2O). We further showed ANXA11 required PI3P to bind liposomes at physiological calcium concentrations (Figures 2P, 2Q). Together, these *in vitro* studies demonstrate that ANXA11 possesses biophysical properties that enable it to interact with both RNA granules and lysosomes, consistent with structural predictions and unbiased proteomic results.

### ANXA11 Interacts with Both RNA Granules and Lysosomes in Cells

**Figure 3:**
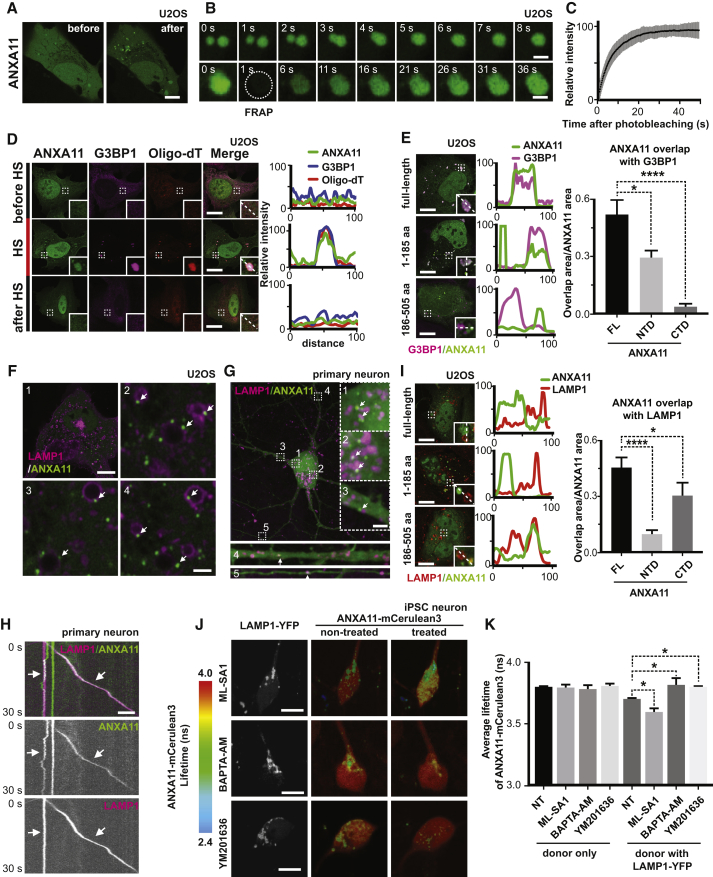
ANXA11 Interacts with Both RNA Granules and Lysosomes in Living Cells (A–D) ANXA11 interact with RNA granules in cells (A) ANXA11-mEmerald redistributes from the cytoplasm into dispersed puncta immediately following heat shock (43^o^C) in U2OS cells. Scale bar: 20 μm. See also Figure S3A. (B) Heat shock induced ANXA11-mEmerald puncta in U2OS cells are motile and undergo fusion (upper panel), and recover rapidly after photobleaching (i.e., FRAP)(bottom panel). Scale bar: 1 μm. See also Figure S3B. (C) Quantification of FRAP experiment in (B), n=23. Error bars = SEM. See also Figure S3C. (D) Immunostaining of mEmerald-tagged ANXA11 with RNA granule markers (Cy3-Oligo dT(30), anti-G3BP1) before, during and 4 hours after heat shock (HS) in U2OS cells. Line scans show the related intensity profiles of ANXA11 with mRNA (Cy3 Oligo-dT) and with G3BP1. Scale bar: 30 μm. See also Figure S3D. (E) Immunostaining of mEmerald-tagged ANXA11 full-length, N-terminal or C-terminal domain and G3BP1 following 30 minutes of heat shock (43^o^C) in U2OS cells. Line scans show the related intensity profiles of ANXA11 with G3BP1. Scale bar: 30 μm. Right panels show the quantification of ANXA11 tructation area overlap with G3BP1(relative to ANXA11 area). One-way ANOVA, ^∗^p < 0.05, ^∗∗∗∗^p < 0.0001, n=10. Error bars = SEM. Scale bar: 30 μm. See also Figures S3E, S3F. (F–K) ANXA11 puncta interact with lysosomes in cells (F) Panel 1: Live cell imaging of U2OS cells expressing ANXA11-mEmerald and LAMP1-HaloTag following heat shock at 43^o^C in U2OS cells. Panels 2-4: Enlarged areas of panel 1, with arrows pointing to ANXA11/lysosome contact sites (Scale bar: 1 μm). See also Figure S3H. (G) Rat cortical neurons expressing LAMP1-HaloTag and ANXA11-mEmerald imaged after heat shock. ANXA11 puncta co-localized with lysosomes in different neuronal regions: soma (1,2), dendrite (3,4) and axon (5). Arrows point to contact sites between lysosomes and ANXA11 puncta. Scale bar: 5 μm (H) Live imaging of rat cortical neuron axons expressing LAMP1-HaloTag and ANXA11-mEmerald. The corresponding kymograph shows ANXA11 puncta (green) either co-trafficking or co-localizing with lysosomes (magenta). Scale bar: 5 μm. See also Figure S3G. (I) Immunostaining of mEmerald-tagged ANXA11 full-length, N-terminal or C-terminal domain with LAMP1-positive lysosomes in U2OS cells following 30 minutes of heat shock (43^o^C). Line scans show related intensity profiles of ANXA11 and LAMP1. Scale bar: 30 μm. Far right panel shows the quantification of ANXA11 trucations and LAMP1 co-localization (relative to ANXA11 area). One-way ANOVA, ^∗^p < 0.05, ^∗∗∗∗^p < 0.0001, n=10. Error bars = SEM. Scale bar: 30 μm. (J) FLIM-FRET analysis of the interaction between ANXA11 and lysosomes and its regulation by lysosomal Ca^2+^ and PI(3,5)P_2_. Human i^3^Neurons were transduced with ANXA11-mCerulean3 (FRET donor) and LAMP-YFP (FRET acceptor). FLIM-FRET images were acquired for the same neurons before and after treatment with ML-SA1, BAPTA-AM or YM201636, and the lifetime of the ANXA11-mCerulean3 signal was determined. Left vertical panels show intensity images of LAMP1-YFP with the various treatments. Middle and right panels show ANAX11-mCeurlean3 lifetimes before and after drug treatment. (K) Quantification of FLIM-FRET lifetime measurements from (*H*). N=31 (NT), 24 (ML-SA1), 20 (BAPTA-AM), 24 (YM201636). One-way ANOVA, ^∗^p < 0.05. Error bars = SEM.

**Figure S3:**
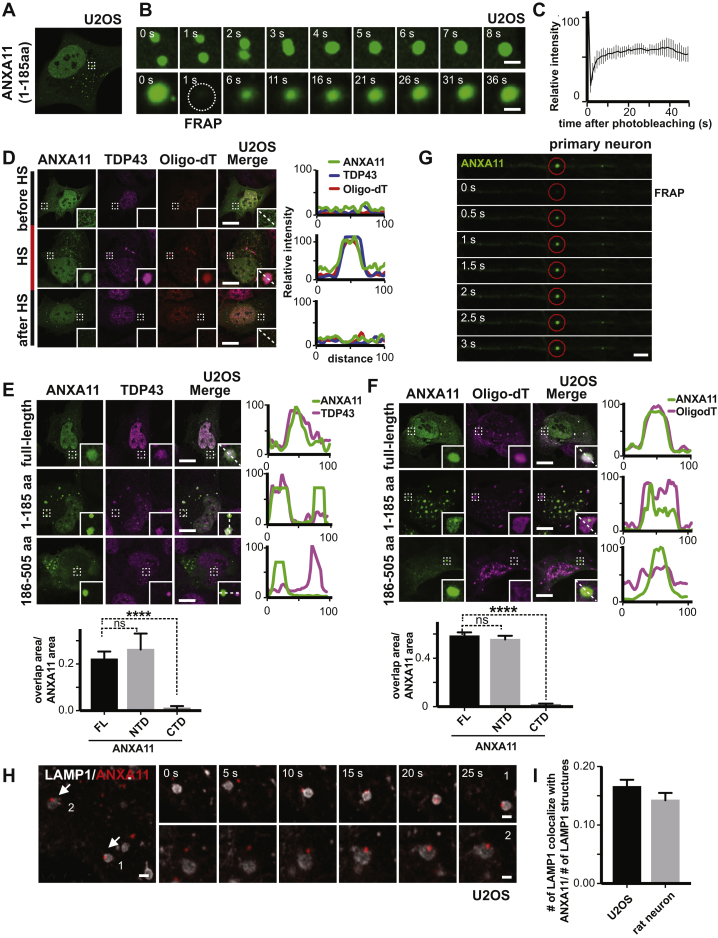
ANXA11 Exhibits Phase Condensate Properties and Interacts with Both RNA Granules and Lysosomes in Living Cells, Related to Figure 3 (A) ANXA11’s amino acid sequence 1-185 was tagged with mEmerald and expressed in U2OS cells. Small ANXA11 positive puncta appeared in cells that had not been heat shocked. (B) Live cell imaging of puncta from (*A*) reveal these structures are motile and undergo fusion (upper panel). Upon photobleaching, ANXA11 fluorescence within the puncta quickly returned from free cytoplasmic pools (bottom panel). Scale bar: 1 μm. (C) Quantification of the FRAP experiment in (*B*), n=7. (D) Co-localization of ANXA11 puncta with RNA granule markers before, during and after heat shock (HS). U2OS cells under normal culture conditions (before HS), under heat shock (HS) or 4 hours after heat shock (after HS) were fixed, hybridized with Cy3-Oligo dT(30) followed by immunostaining with antibody against TDP43. Linescans show the related intensity profiles of ANXA11 with mRNA (Cy3 Oligo-dT) and with TDP43. Scale bar: 30 μm. (E) Co-localization of ANXA11 full-length, N-terminal or C-terminal domain with RNA granules. U2OS cells were fixed after 30 minutes of heat shock (43^o^C), followed by immunostaining with antibody against TDP43. Line scans show the related intensity profiles of ANXA11 with TDP43. Scale bar: 30 μm. One-way ANOVA, ^∗∗∗∗^p < 0.0001, n=20 (FL), 11(NTD), 19(CTD), Error bars=SEM. (F) Co-localization of ANXA11 full-length, N-terminal or C-terminal domain with RNA granule. U2OS cells were fixed after 30 minutes of heat shock (43^o^C), followed by hybridizing with Cy3-Oligo dT(30). Linescans show the related intensity profiles of ANXA11 with TDP43. Scale bar: 30 μm. One-way ANOVA, ^∗∗∗∗^p < 0.0001, n=30 (FL), 30(NTD), 27(CTD), Error bars=SEM. (G) Rat cortical neurons were transduced with ANXA11-mEmerald. A single labeled ANXA11 puncta was photobleached, then the recovery of fluorescence into the bleached region-of-interest was examined over time. Scale bar: 2 μm. (H) Time-lapse imaging showing the interaction of ANXA11 puncta (red) with LAMP1-labeled lysosomes (white) in U2OS cells after heat-shock. Scale bar: 1 μm. (I) Quantification of LAMP1 labeled lysosomes co-localizing with ANXA11(relative to number of lysosomes) in U2OS or rat neuron. N=25(U2OS), 10(neuron).

Based on its structural and biophysical attributes, we speculated that ANXA11 might incorporate into RNA granules through its phase separating properties and additionally interact with lysosomes through its lipid binding properties. Basic characteristics of phase-separated RNA granules in cells include dynamic structural associations (i.e., fission and fusion), rapid exchange between phase-separated and soluble states, and stress-induced oligomerization (i.e., “stress granule” formation) (Hyman and Brangwynne, 2011, Hyman et al., 2014). We found that ANXA11-mEmerald redistributed into spheroid structures following heat shock (Figure 3A). These stress-induced structures had various liquid properties, including droplet fusion (Figure 3B, top panel) and rapid fluorescence recovery after photobleaching (Figure 3B, bottom panel, and Figure 3C), the latter indicating rapid cycling of ANXA11 between phase-separated and soluble states. The N-terminal LC region of ANXA11 was sufficient for ANXA11 puncta formation (Figures S3A–S3C), consistent with its *in vitro* properties.

We then asked if ANXA11 co-localized with RNA stress granule markers in cells undergoing heat shock. Stress granules composed of G3BP1, TDP43 or mRNA labeled by Oligo-dT all contained ANXA11 signal (Figure 3D, Figure S3D). Because the N-terminal LCR of ANXA11 conferred its phase separation properties, we speculated that this region also mediated interactions with RNA granules in cells. We found that the N-terminal LCR of ANXA11 was sufficient to localize mEmerald to stress granules in U2OS cells (albeit to a lower extent than full-length ANXA11), but that an ANXA11-mEmerald truncation mutant lacking the N-terminal LCR was not (Figure 3E, Figures S3E and S3F). These data indicate that ANXA11 incorporates into stress-induced RNA granules following heat shock, that these granules represent heterogeneous, phase-separated assemblies, and that the N-terminal LCR of ANXA11 is necessary and sufficient for RNA granule interactions.

We next examined whether ANXA11 interacted with lysosomes in living cells. Time-lapse imaging revealed that ANXA11-positive puncta localized to the surface of LAMP1-structures following heat shock in U2OS cells (Figure 3F and Figure S3H) and primary neurons (Figure 3G, Figure S3G). Axonal ANXA11 puncta co-trafficked with lysosomes, as shown in kymographs (Figure 3H). As predicted by our structural modeling, the C-terminal annexin repeat domain was both necessary and sufficient for interactions between ANXA11 and lysosomes (Figure 3I). Taken together, these results indicate that ANXA11 interacts with both RNA granules and lysosomes within diverse cell types, and that these interactions are mediated by its N-terminal LCR and C-terminal annexin domains, respectively.

To further explore the interactive properties of lysosomes and ANXA11 in living cells, we used a FLIM-based FRET approach, which can be used to infer direct molecular interaction between two probes at nanometer scales. We found that the lifetime of the FRET donor ANXA11-mCerulean3 decreased (i.e., FRET efficiency increased) near the FRET acceptor LAMP1-YFP, suggesting that ANXA11 and lysosomes tightly associate with each other (Figure 3J). Interestingly, the FRET efficiency increased further in the presence of ML-SA1, a lysosomal calcium channel agonist (Figures 3J and 3K), suggesting that ANXA11 and lysosomes more strongly interact following Ca^2+^ release from lysosomes. Supporting this observation, treatment with BAPTA-AM, a selective, permeable Ca^2+^ chelator that removes free Ca^2+^ from the cytoplasm, decreased the FRET efficiency (Figures 3J and 3K). Treatment with YM201636, which inhibits the formation of PI(3,5)P_2_, also decreased the FRET efficiency, suggesting that ANXA11 and lysosomes depend on PI(3,5)P_2_ for their interaction (Figures 3J and 3K). Therefore, the interaction between ANXA11 and lysosomal membranes in cells occurs in a calcium- and phospholipid- dependent manner.

### ALS-Associated Mutations in ANXA11 Disrupt RNA Granule Dynamics and Interactions

**Figure S4:**
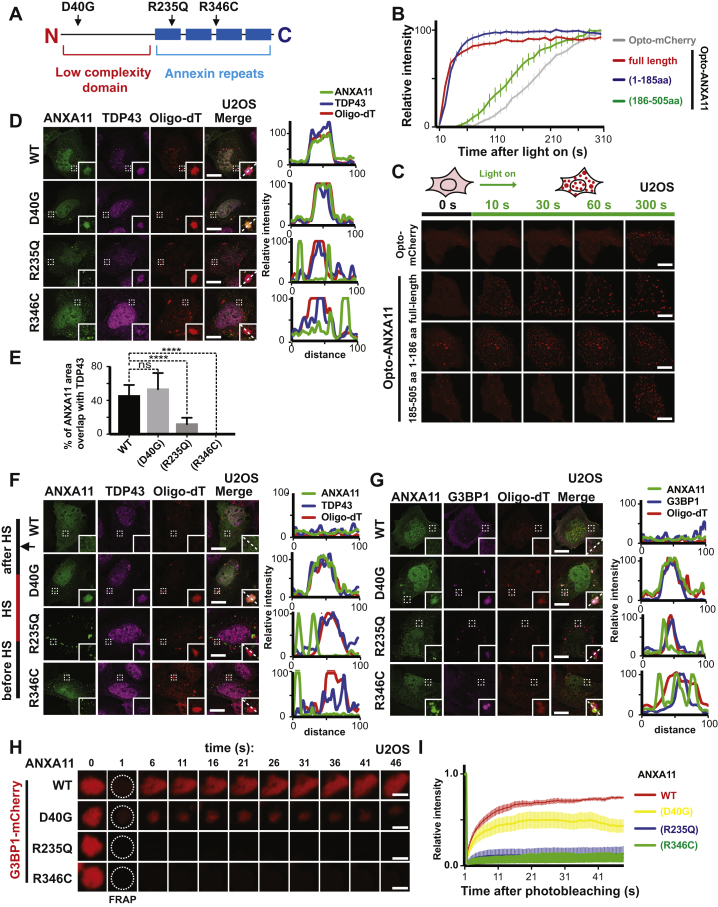
ALS-Associated Mutations in ANXA11 Disrupt RNA Granule Interactions, Related to Figure 4 (A) A schematic map of ANXA11 protein with the position of ALS-associated mutants. (B) Quantification show the temporal evolution of the integrated fluorescence intensity from the expressed Opto-mCherry, ANXA11 full-length, NTD or CTD proteins during 300 seconds of light activation, n=11 (Opto-mCherry), 17 (ANXA11 full-length), 20(ANXA11 NTD), 20(ANXA11 CTD). Error bars = SEM. (C) U2OS were transfected with Opto-mCherry (CRY2olig-mcherry), Opto-ANXA11, Opto-ANXA11 NTD or Opto-ANXA11 CTD for 24hrs. Cells with similar Opto-ANXA11 expression levels were exposed to 0.2% 488nm light to initiate oligomerization. Scale bar: 30 μm. (D) Co-localization of ANXA11 or ANXA11 ALS-associated mutants with TDP43 and mRNA labeled by Oligo-dT. U2OS cells expressing mEmerald labeled ANXA11 or ANXA11 ALS-associated mutants were heat shocked for 30 mins, fixed, and then hybridized with Cy3-Oligo dT(30) and immunostained with antibodies against TDP43 to label RNA granules. The extent of co-localization of ANXA11 or the ALS-associated mutants with the RNA granules is plotted in the line-scans to the right. (E) Quantification of percentage of area of ANXA11 structures co-localizing with TDP43-labeled RNA granules in (*D*). One-way ANOVA, ns, not significant. ^∗∗∗∗^p < 0.0001. Error bars = SEM. N=12 (WT), 7 (D40G), 6 (R235Q), 7(R346C). (F) Co-localization of ANXA11 or ANXA11 ALS-associated mutants with RNA granules labeled by TDP43 and mRNA labeled by Oligo-dT after heat shock (HS). U2OS were heat shocked for 30 mins and then moved to 37^o^C for 4 hrs to allow recovery. The cells were then fixed, hybridized with Cy3-Oligo dT(30) followed by immunostaining with antibodies against TDP43 to label RNA granules. Linescan analysis show the related intensity profiles of ANXA11 or ALS-associated mutations with mRNA (Cy3 Oligo-dT) and TDP43. Scale bar: 30 μm. (G) Co-localization of ANXA11 or ALS-associated ANXA11 mutants with RNA granules labeled by G3BP1 and mRNA (right panel) after heat shock (HS). U2OS were heat shocked for 30 mins and then moved to 37^o^C for 4 hrs to allow recovery. The cells were then fixed, hybridized with Cy3-Oligo dT(30) followed by immunostaining with antibodies against G3BP1 (right panel) to label RNA granules. Linescan analysis show the related intensity profiles of ANXA11or ALS-associated ANXA11 mutants with mRNA (Cy3 Oligo-dT) and G3BP1. Scale bar: 30 μm. (H) U2OS cells expressing mCherry-G3BP1 to label RNA granules were co-transfected with ANXA11-mEmerald, ANXA11(D40G)-mEmerald, ANXA11(R235Q)-mEmerald or ANXA11(R346C)-mEmerald for 24 hrs. Cells were heat shocked (43^o^C) for 30 min, A single G3BP1-positive puncta in each of the different transfected cells was photobleached and recovery of fluorescence into the puncta was monitored by time-lapse imaging. Scale bar: 1 μm. (I) Quantification of H. N=7(WT), 9(D40G), 8(R235Q), 7(R346C). Error Bars=SEM.

**Figure 4:**
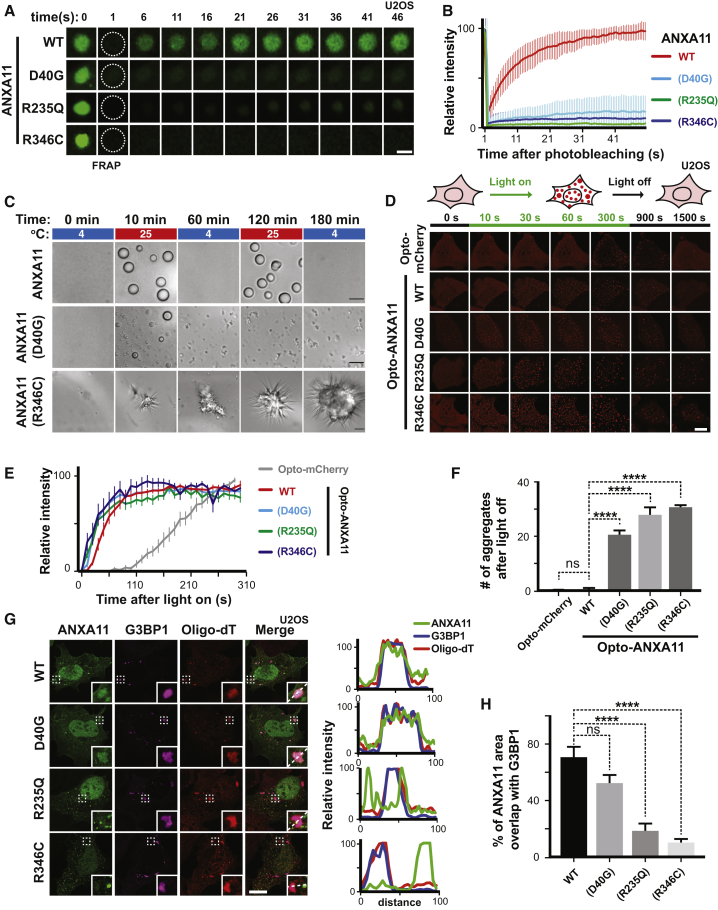
Effects of ALS-Associated ANXA11 Mutations on RNA Granule Interactions (A) U2OS cells expressing mEmerald-tagged ANXA11 (WT, D40G, R235Q or R346C) were heat shocked (43^o^C) for 30 minutes. A single ANXA11-positive puncta in each of the different transfected cells was photobleached and recovery of fluorescence was monitored by time-lapse imaging. Scale bar: 1 μm. (B) Quantification of the FRAP experiments in (*A*), n=21. Error bars = SEM. (C) Phase partitioning characteristics of ANXA11 ALS-associated mutants *in vitro*. Purified WT, D40G, R346C forms of ANXA11 were temperature transitioned between 4^o^C and 25^o^C through multiple cycles. ANXA11(p.D40G) formed both large spherical droplets and smaller, non-fusing gelled condensates, with the condensates unable to reform after one round of temperature shift (middle panel). ANXA11(p.R346C) formed irregularly-shaped solid and spiculated gelled condensates with few, if any, liquid droplets capable of disassembly/reassembly during temperature shifts (bottom panel). Scale bar: 5 μm. (D) U2OS cells expressing similar levels of Opto-mCherry (CRY2olig-mCherry), Opto-ANXA11 or Opto-ANXA11 ALS-associated mutant were exposed to 0.2% 488nm light to initiate oligomerization. Scale bar: 30 μm. See also Figure S4B. (E) Quantification of integrated fluorescence intensity of Opto-labeled proteins in *(D)* during 300 seconds of 488 nm light activation, n=20. Error bars = SEM. See also Figure S4C. (F) Quantification of the number of Opto-labeled puncta present 30 minutes after the 488 nm light was turned off. n=17-19. One-way ANOVA, ns, not significant. ^∗∗∗∗^p < 0.0001. Error bars= SEM. (G) Immunostaining of mEmerald-tagged wild-type and mutant ANXA11 with G3BP1 and mRNA labeled by Oligo-dT in U2OS cells following 30 minutes of heat shock. Co-localization of ANXA11 with individual RNA granules is plotted in the line scans to the right. Scale bar: 30 μm. See also Figure S4D. (H) Quantification of area of ANXA11 structures co-localizing with G3BP1-labeled RNA granules in (G). n=28-31. One-way ANOVA, ns, not significant. ^∗∗∗∗^p < 0.0001. Error bars = SEM.

Since mutations in both the N- and C-terminal domains of ANXA11 are associated with familial ALS, we investigated whether these mutations altered properties of RNA granules in living cells. FRAP analysis of ANXA11 dynamics showed that granules containing p.D40G-ANXA11, p.R235Q-ANXA11 or p.R346C-ANXA11 (Figure S4A) had impaired fluorescence recovery relative to WT ANXA11 (Figures 4A and 4B). This result suggests that ALS mutations cause ANXA11 to become more stably associated with RNA granules and/or impair their phase transitioning.

To determine whether this effect was intrinsic to ANXA11, we performed *in vitro* assays using purified WT, p.D40G, and p.R346C forms of ANXA11. We transitioned the temperature between 4^o^C and 25^o^C over repeated cycles and observed the proteins’ ability to phase partition into droplets. Both p.D40G and p.R346C mutants exhibited accelerated phase transitioning from soluble protein to insoluble gels upon warming, and an impaired ability to recover into liquid states upon re-cooling (Figure 4C). Therefore, ALS-associated ANXA11 mutations promote phase transitions from liquid to more stable gel-like states within ANXA11 droplets.

Next, we fused a light-induced oligomerization domain (CRY2-mCherry) to the N terminus of WT and mutant ANXA11 proteins (Opto-ANXA11), allowing us to precisely regulate assembly/disassembly of ANXA11 phase condensates using light. Exposure of wild-type Opto-ANXA11 to 488-nm light triggered oligomerization faster than Opto-mCherry (Figures 4D and 4E), and the N-terminal LCR of ANXA11 was necessary and sufficient for Opto-ANXA11 condensation (Figures S4B and S4C). Thus, ANXA11 potentiates light-induced phase condensation, similar to other RNA-granule proteins fused with CRY2 (Shin et al., 2017, Zhang et al., 2018b). All mutant Opto-ANXA11 proteins tested formed condensates faster than WT-Opto-ANXA11 (Figures 4D and 4E). Moreover, mutant Opto-ANXA11 condensates disassembled substantially slower than WT-Opto-ANXA11 following discontinuation of 488-nm light stimulation (Figures 4D and 4F). Together, these results suggest that N- and C-terminal ALS-associated ANXA11 mutations promote phase transitions from liquid-liquid droplets to gel-like states, and impair reversal of gel-like states once formed.

To test whether mutations in ANXA11 altered its ability to interact with RNA granules, we quantified the extent of co-localization of ANXA11 and its mutants with RNA granules following heat shock in living cells. Whereas the N-terminal p.D40G-ANXA11 mutation had no effect on RNA granule co-localization, C-terminal p.R235Q-ANXA11 and p.R346C-ANXA11 mutations dramatically reduced co-localization of ANXA11 with RNA granules (Figure 4G, H, Figures S4D and S4E). ALS-associated ANXA11 mutations also altered the phase transition properties of other RNA granule-associated proteins, slowing both FRAP kinetics of G3BP1 and the disassembly rate of G3BP1 granules following stress release (Figures S4F–S4I). Thus, ALS-associated mutations in ANXA11 interfere with ANXA11’s ability to interact and intercalate with phase-separated RNA granules, which subsequently impacts RNA granule properties.

### ALS-Associated Mutations in ANXA11 Disrupt Its Interactions with Lysosomes

**Figure 5:**
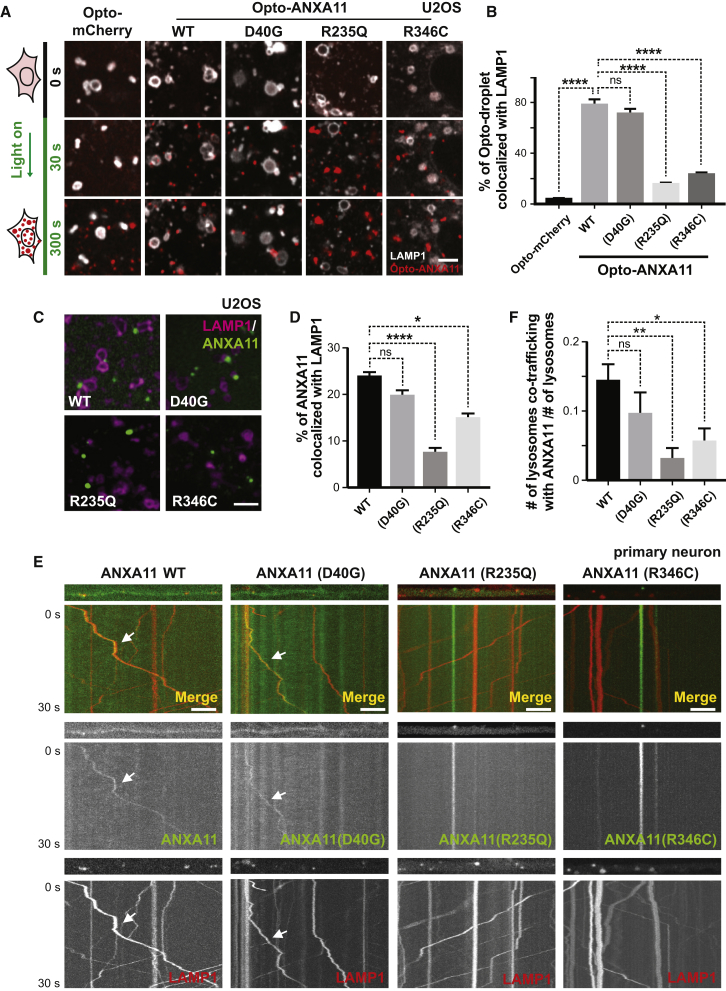
ALS-Associated Mutations in ANXA11 Disrupt Its Interactions with Lysosomes (A) Live cell imaging of Opto-mCherry, wild-type Opto-ANXA11, or mutant Opto-ANXA11 with LAMP1-HaloTag in U2OS cells before and after oligomerization induced by exposure to 488nm light. Scale bar: 2 μm. See also Figure S5A. (B) Quantification of percentage of light-activated Opto-mcherry (CRY2olig-mCherry), wild-type Opto-ANXA11, or mutant Opto-ANXA11 clusters co-localizing with lysosomes at 300s post-488 nm light exposure from (*A*). n=26-30. One-way ANOVA, ns, not significant. ^∗∗∗∗^p < 0.0001. Error bars = SEM. See also Figure S5B. (C) Extent of co-localization of wild-type or mutant ANXA11 with lysosomes. U2OS cells expressing LAMP1-HaloTag, wild-type ANXA11-mEmerald or mutant ANXA11-mEmerald were imaged 30 minutes after heat shock (43^o^C). Scale bar: 2 μm. (D) Percentage of fluorescence associated with wild-type ANXA11 or mutant ANXA11 that co-localized with lysosomes from (*C*). n= 22-40. One-way ANOVA, ns, not significant. ^∗∗∗∗^p < 0.0001. ^∗^p < 0.05. Error bars = SEM. (E) Extent of co-trafficking of wild-type or mutant ANXA11 with lysosomes in axons. Axons of rat cortical neurons expressing LAMP1-HaloTag and wild-type or mutant ANXA11-mEmerald were imaged for 30 seconds. Kymographs show WT and p.D40G ANXA11 both co-traffic with lysosomes (see arrows) while p.R235Q and p.R346C each disrupt ANXA11 co-trafficking with lysosomes. Scale bar: 10 μm. (F) Number of puncta containing WT or mutant ANXA11 that co-trafficked with lysosomes as a function of total lysosome number from (*E*). n= 21-30. One-way ANOVA, ns, not significant. ^∗∗^p < 0.01. ^∗^p < 0.05. Error bars = SEM.

**Figure S5:**
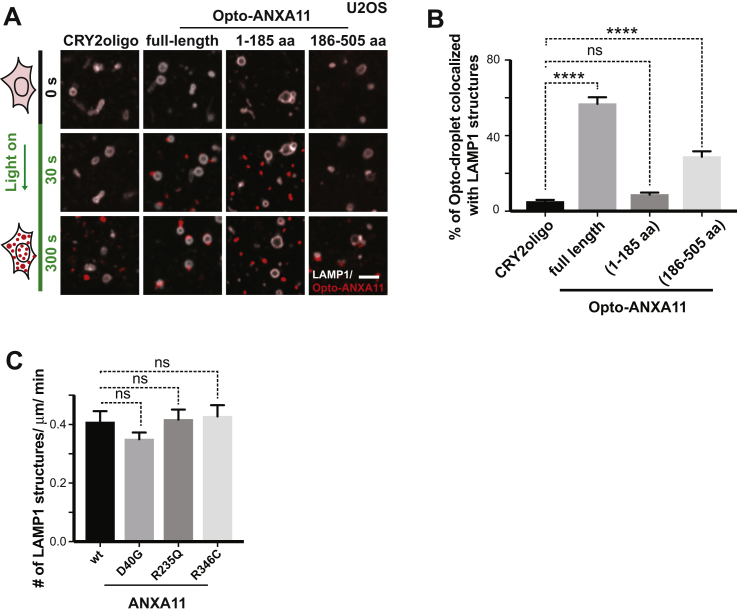
ALS-Associated Mutations in ANXA11 Disrupt Its Interactions with Lysosomes, Related to Figure 5 (A) Co-localization of light-activated opto-ANXA11 or ANXA11 N-terminal domain or C-terminal domain with lysosomes in cells. U2OS cells were co-transfected with LAMP1-HaloTag, Opto-mcherry (CRY2olig-mcherry), Opto-ANXA11, Opto-ANXA11 NTD or Opto-ANXA11 CTD for 24 hrs. Cells with similar Opto-ANXA11 expression levels were exposed to 0.2% 488nm light to initiate oligomerization. Cells were imaged over 300 seconds of light activation. Scale bar: 2 μm. (B) Percentages of light-activated Opto-mcherry (CRY2olig-mcherry), Opto-ANXA11 Opto-ANXA11 NTD or Opto-ANXA11 CTD clusters co-localizing with lysosomes after 300 seconds of light activation from the experiment in (*A*). n=14 (Opto-mcherry), 18 (ANXA11 full-length), 21(ANXA11 NTD), 21(ANXA11 CTD). One-way ANOVA, ns, not significant. ^∗∗∗∗^p < 0.0001. Error bars = SEM. (C) Frequency of LAMP1 labeled vesicles in axons expressed ANXA11 or ALS-associated ANXA11 mutants. n=25(WT), 50(D40G), 15(R235Q), 22(R346C). One-way ANOVA, ns, not significant. Error bars = SEM.

We next asked whether ALS-associated ANXA11 mutations altered lysosome interactions. Light-induced oligomerization of Opto-ANXA11 caused rapid association of Opto-ANXA11 condensates with lysosomes (Figures 5A and 5B). The C-terminal annexin-repeat domain of ANXA11 was necessary and sufficient for Opto-ANXA11 association with lysosomes (Figures S5A and S5B). Notably, Opto-ANXA11 mutants harboring ALS-associated C-terminal mutations failed to interact with lysosomes (Figures 5A and 5B). C-terminal ALS-associated mutations also impaired associations between ANXA11-mEmerald condensates and lysosomes following stress (Figures 5C and 5D). Therefore, as predicted by structural modeling, ANXA11 interacts with lysosomes through its C-terminal domain, a process disrupted by ALS-associated C-terminal mutations.

We further tested whether ALS-associated mutations altered axonal co-trafficking of ANXA11 with lysosomes in cultured primary neurons. As predicted, mutations in the C terminus of ANXA11 impaired ANXA11’s ability to interact with motile lysosomes in axons (Figures 5E and 5F) while having no substantial effect on lysosome trafficking (Figure S5C). Taken together, these data suggest that ALS-associated C-terminal mutations impair the ability of ANXA11 to associate with lysosomes, and both C- and N-terminal mutations impact properties of ANXA11 within phase-separated structures.

### ANXA11 Acts as an Adaptor between RNA Granules and Lysosomes

**Figure 6:**
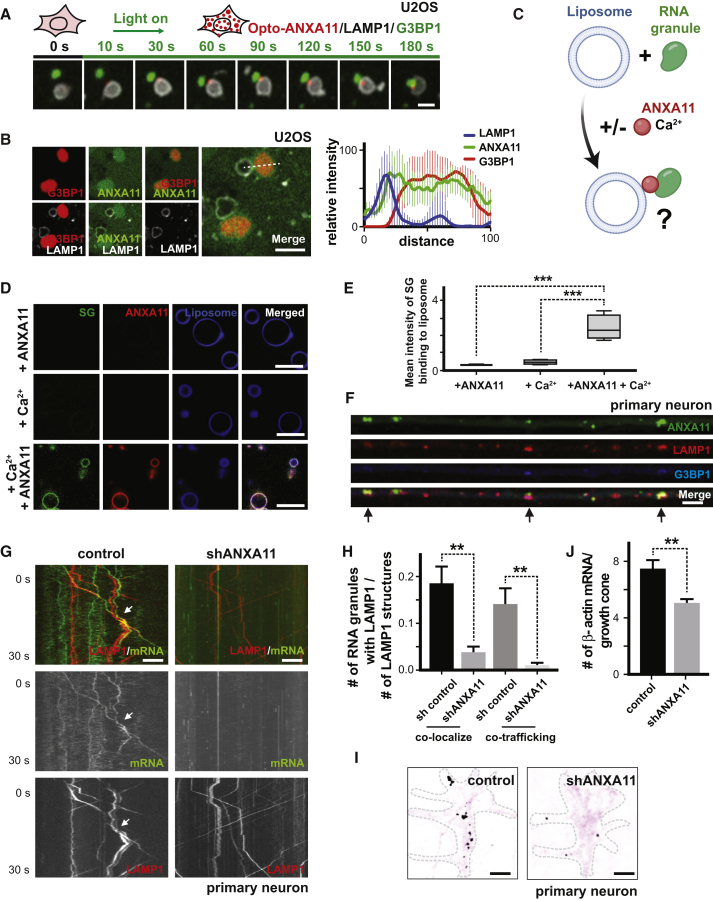
ANXA11 Acts as an Adaptor between RNA Granules and Lysosomes (A) Time-lapse imaging of U2OS cells expressing LAMP1-HaloTag, Opto-ANXA11 and mEmerald-G3BP1 after 488nm light exposure to induce Opto-ANXA11 oligomerization. U2OS cells were exposed to heat shock (43 ^o^C) for 15 minutes prior to light activation to form visible G3BP1 stress granules. Stress granules (green) associate with LAMP1-labeled lysosomes (white) at sites where ANXA11 puncta (red) are localized. Scale bar: 1 μm. See also Figure S6A, Video S3. (B) Live cell confocal imaging of U2OS expressing LAMP1-HaloTag, ANXA11-mEmerald and mCherry-G3BP1 following 30 minutes of heat shock (43^o^C). Quantification of the intensity profiles of the different probes across midline of stress granules (dotted line) is shown to right. n=6, Error bars = SEM. Scale bar: 1μm. (C) Schematic of *in vitro* RNA granule liposome reconstitution assay. (D) Stress granule cores were purified from cultured cells, and incubated with PI3P containing liposomes +/– recombinant ANXA11 +/– Ca^2+^. Upper panel: + ANXA11 only, Middle panel: + Ca^2+^only, Bottom panel: + both ANXA11 and Ca^2+^. Scale bar=10 μm. (E) Quantification of mean intensity of stress granule binding to PI3P containing liposomes in *(D)*. n>300, One-way ANOVA, ^∗∗∗^p < 0.001. Error bars = SEM. (F) Co-localization of ANXA11, lysosomes, and RNA granules in axons. Rat cortical neurons were transduced with LAMP1-HaloTag to label lysosomes, ANXA11-mEmerald to label ANXA11, and mCherry-G3BP1 to label RNA granules. Arrows indicate areas of ANXA11, lysosome and RNA granule co-localization. Scale bar: 5 μm. See also Figure S7A, Video S4, 5. (G) ANXA11 knockdown perturbs mRNA/lysosome co-trafficking in axons. Kymographs of mRNA (actin-24xMBS/ MCP-NLS-2xEGFP) and lysosome (LAMP1-HaloTag) trafficking in axons is shown. Rat neurons expressed control or ANXA11-targeting shRNAs. Scale bar: 5 μm. (H) Quantification of *(G)*. n=22-36, t-test, ^∗∗^p < 0.01. Error bars = SEM. (I) smFISH of beta-actin in growth cones from neuron expressing control shRNA (left panel) or ANXA11 shRNA (right panel). Black colored spots represent the signal from beta-actin smFISH probes, red signal represents membrane stain of growth cones. Scale bar: 1 μm. (J) Quantification of average number of beta-actin mRNA molecules in *(I)*. N=44-96, t-test, ^∗∗^p < 0.01. Error bars = SEM.

**Figure S6:**
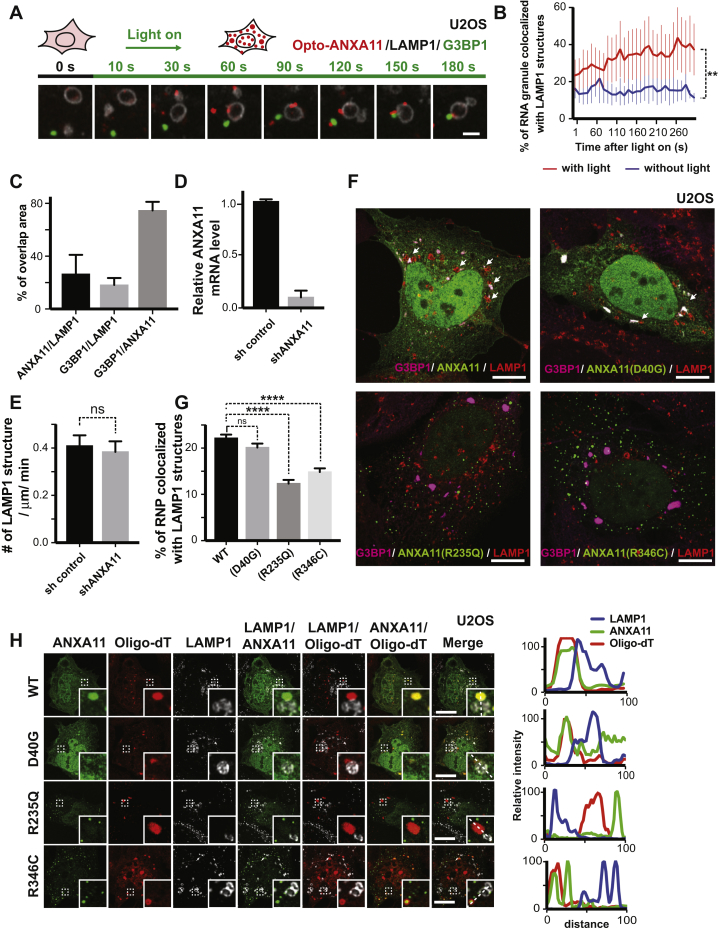
ANXA11 Acts as an Adaptor between RNA Granules and Lysosomes, Related to Figure 6 (A) Additional example of a time-lapse imaging sequence of U2OS cells expressing LAMP1-HaloTag, Opto-ANXA11 and mEmerald-G3BP1 after 0.2% 488nm light activation to initiate Opto-ANXA11 oligomerization. U2OS cells were exposed to heat shock (43 ^o^C) for 15 minutes prior to light activation. Here, G3BP1-labeled RNA granules (green) associate with ANXA11 puncta (red) in the cytoplasm before redistributing onto the surface of LAMP1-labeled lysosomes (white) as merged puncta. Scale bar: 1 μm. (B) Quantification of the percentage of RNA granules co-localizing with LAMP1 over 300s with or without light activation in (A). N=11. Paired t-test, ^∗∗^, p<0.01. Error bars = SEM. (C) Percentage of co-localization between ANXA11 and LAMP1, G3BP1 and LAMP1, G3BP1 and ANXA11. N=20. Error bars = SEM. (D) Q-PCR shows relative ANXA11 mRNA level in control shRNA or ANXA11 shRNA transduced neurons. N=3. Error bars = SEM. (E) Frequency of LAMP1-labeled vesicles in axons expressed control shRNA or ANXA11 shRNA. N=36(sh control), 35 (sh ANXA11), t-test, ns, not significant. Error bars = SEM. (F) Effect of ALS-associated ANXA11 mutants on RNA granule-lysosome association in heat shocked cells. U2OS cells were transfected with mEmerald tagged ANXA11 or ANXA11 ALS-related mutant constructs. Cells were heat shocked (43^o^C) for 30 minutes, fixed, followed by immunostaining with antibodies against G3BP1 and LAMP1 to examine the effects of ANXA11 mutants on RNA granule-lysosome contacts compared to those in WT ANXA11-mEmerald expressing cells. Arrows point to G3BP1-labeled RNA granule(megenta) contacting with lysosomes(red). Scale bar: 30 μm. (G) Percentage of G3BP1-labeled granules co-localized with LAMP1-labeled lysosomes in (*E*). n= 20 (WT), 25 (D40G), 28 (R235Q), 28 (R346C). One-way ANOVA, ns, not significant. ^∗∗∗∗^p < 0.0001. Error bars = SEM. (H) U2OS cells were transfected with mEmerald tagged ANXA11 or ANXA11 ALS-related mutant constructs. Cells were heat shocked (43^o^C) for 30 minutes, fixed, hybridized with Cy3-Oligo dT(30) followed by immunostaining with antibodies against LAMP1 to examine the effects of ANXA11 mutants on RNA granule-lysosome contacts compared to that in WT ANXA11-mEmerald expressing cells. Graphs on the right represent intensity profiles across the dotted line, revealing the ANXA11 mutants R235Q and R346C show decreased colocalization with RNA granules and lysosomes and affected RNA granule-lysosome contact. Scale bar: 1μm.

Since ANXA11 interacts with both RNA granules and lysosomes, we speculated that ANXA11 might function as a molecular tether to couple RNA granules with lysosomes. To explore this possibility, we expressed Opto-ANXA11 in U2OS cells and monitored lysosome and RNA granule dynamics using time-lapse confocal microscopy. We reasoned light-induced oligomerization of ANXA11 might facilitate the docking of RNA granules with lysosomes, since phase-separated ANXA11 has an increased affinity for both structures. When we stimulated Opto-ANXA11 expressing cells with 488 nm light, we saw G3BP1-labeled RNA granules associating with Opto-ANXA11-bound lysosomes (Figure 6A, Figure S6A, Video S3) and an increase in the number of RNA granule-lysosome interactions (Figure S6B).

Video S3. Opto-ANXA11 associated with RNA granules and lysosomes. Related to Figure 6

We then co-imaged ANXA11, RNA granules and lysosomes in heat-shocked U2OS cells to further define their spatial relationships. ANXA11 was present in the core of G3BP1-labeled RNA granules and additionally displayed a peripheral localization pattern that extended beyond the boundary of G3BP1 into the region labeled by LAMP1 (Figure 6B), consistent with a potential tethering function.

Next, we asked if ANXA11 was sufficient to promote interactions of RNA granules with lysosome-like vesicles in an *in vitro* reconstitution assay. We purified stress-induced RNA granule cores from cells (Jain et al., 2016, Khong et al., 2017, Wheeler et al., 2017), and mixed these granules with PI3P-containing liposomes in the presence or absence of ANXA11 and/or calcium (Figure 6C). In the absence of ANXA11 or calcium, G3BP1-positive RNA granules failed to interact with liposomes. However, addition of both ANXA11 and calcium promoted contact between G3BP1-positive RNA granules and liposomes (Figures 6D and 6E). These results support a model in which ANXA11 directly functions as a molecular tether to facilitate binding of RNA granules to lysosomes.

To determine if ANXA11 co-localized with co-motile RNA granules and lysosomes in axons, we performed time-lapse imaging of cultured rodent neurons. Similar to stress granules, ANXA11 co-localized and co-trafficked with motile RNA granule/lysosome assemblies in axons (Figure 6F, Video S4).

**Figure 7:**
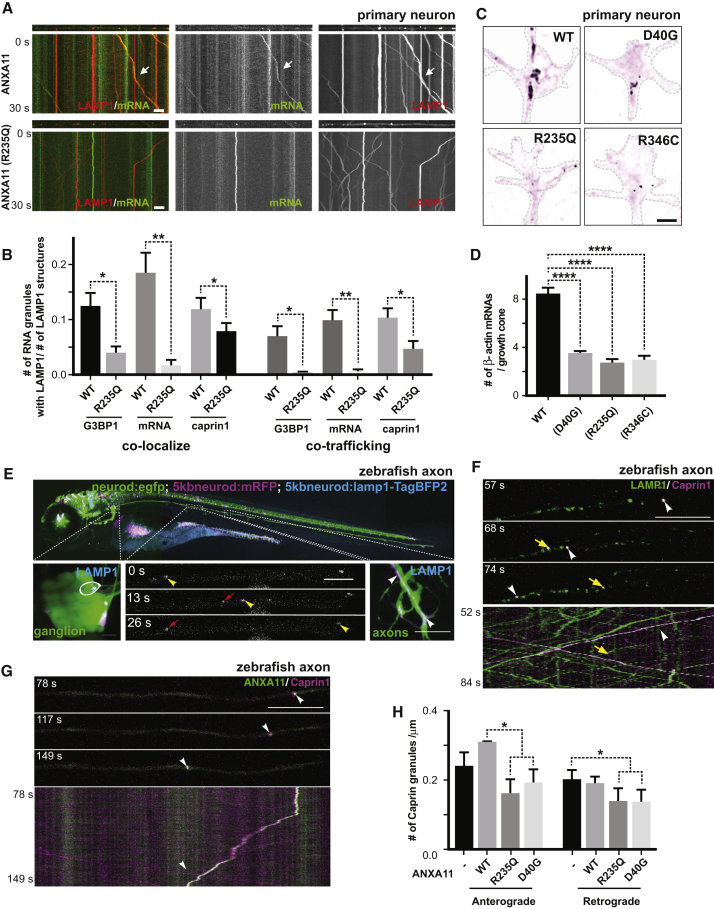
Effects of ALS-Associated ANXA11 Mutations on Axonal RNA Granule/Lysosome Hitchhiking (A) Kymographs of mRNA (actin-24xMBS/ MCP-NLS-2xEGFP ) and lysosome (LAMP1-HaloTag) trafficking in rat neuron axons expressing wild-type or R235Q mutant ANXA11. Arrows point to examples of mRNA co-trafficking with lysosomes. Scale bar: 10 μm. (B) Quantification of *(A)*. n=14-36. One-way ANOVA. ^∗^p < 0.05, ^∗∗^p < 0.01. Error bars = SEM. (C) smFISH of beta-actin in growth cones from rat neurons expressing wild-type or mutant ANXA11. Black colored spots represent the signal from beta-actin smFISH probes, red signal represents membrane stain of growth cones. Scale bar: 1 μm. (D) Quantification of *(C)*. N= 64-128. One-way ANOVA. ^∗∗∗∗^p < 0.0001, Error bars = SEM. (E) Lysosome trafficking in live zebrafish embryo ganglion axons. Lysosomes were labeled with LAMP1-TagBFP2 in zebrafish pLL ganglions; insets show ganglion (left) and axon tips (right). Time-lapse imaging reveals bi-directional lysosomal trafficking in these axons (bottom middle panels). (F) Imaging of live zebrafish neurons reveals bi-directional co-trafficking of CAPRIN1-positive RNA granules with lysosomes in axons. Yellow arrows point to anterograde co-trafficking of LAMP1 (green) and CAPRIN1 (magenta); white arrows point to retrograde co-trafficking of LAMP1 (green) and CAPRIN1 (magenta). Corresponding kymograph shown below. See also Video S6. (G) Imaging of live zebrafish neurons expressing ANXA11 and CAPRIN1 reveals co-trafficking of ANXA11-labeled structures (green) with CAPRIN1 (magenta) in axons. Corresponding kymograph shown below. See also Video S7. (H) Effect of ANXA11 ALS-associated mutations on trafficking of CAPRIN1-labeled RNA granules in zebrafish axons. CAPRIN1 and wild-type or mutant ANXA11 were expressed in zebrafish ganglion. Anterograde or retrograde trafficking of CAPRIN1 vesicles per μm along the axon length were quantified in each group. n = 9-18. Two-way ANOVA with Tukey post-hoc analysis, ^∗^p < 0.05. Error bars = SEM. See also Figure S7E-S7G.

Video S4. RNA granule co-trafficking with lysosomes and ANXA11 in primary rat neuron axons. Related to Figure 7

We then asked if ANXA11 was necessary to facilitate axonal RNA granule/lysosomal hitchhiking. ANXA11 knockdown in primary rodent neurons (Figure S6D) substantially impaired axonal RNA granule/lysosome hitchhiking (Figures 6G and 6H) without altering axonal lysosome transport itself (Figure S6E). To determine if ANXA11 knockdown impaired RNA delivery to distal regions of the cell, we quantified levels of actin mRNA in growth cones using single molecule FISH (smFISH). We found that ANXA11 knockdown reduced levels of actin mRNA in growth cones, consistent with impaired long-distance axonal mRNA transport (Figures 6I and 6J). Together, these data indicate that ANXA11 is sufficient to facilitate tethering of RNA granules to lysosomes, and is necessary for axonal RNA granule hitchhiking and delivery of mRNA to distal locations within neurons.

### ALS-Associated ANXA11 Mutations Disrupt RNA Granule Hitchhiking on Lysosomes

Because C-terminal mutations interfered with the ability of ANXA11 to interact with lysosomes, we tested whether these mutations disrupted RNA granule-lysosome interactions. Indeed, lysosomes made fewer contacts with RNA granules in cells expressing ANXA11 with C-terminal mutations (Figures S6F–S6H). These findings predict that ALS-associated ANXA11 mutations, which interfere with the ability of ANXA11 to efficiently interact with RNA granules and lysosomes, also disrupt hitchhiking of RNA granules on lysosomes during transport.

**Figure S7:**
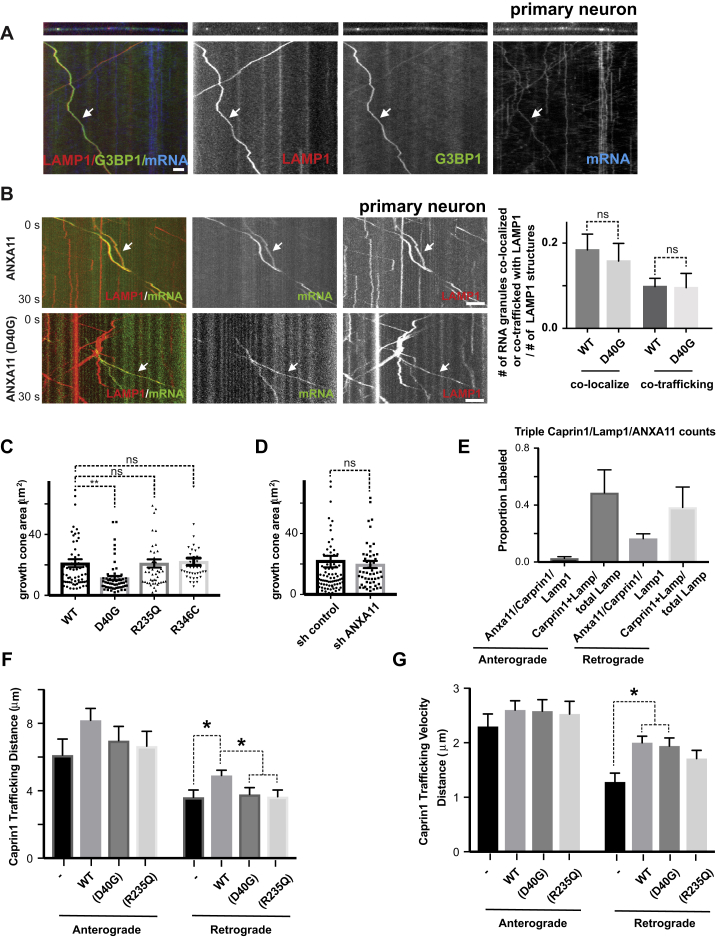
ALS-Associated Mutations in ANXA11 Disrupt RNA Granule Hitchhiking on Lysosomes in Axons from Rat Cortical Neurons and Zebrafish Neurons, Related to Figure 7 (A) Kymographs showing RNA granule protein and mRNA co-transport on the same lysosomes in axons. Rat cortical neurons were transduced with LAMP1-HaloTag to label lysosomes (red), and mCherry-G3BP1 to label RNA granules (green), and actin-24xMBS/ MCP-NLS-2xEGFP(blue). Time-lapse movies of axons were then acquired at 100ms/frame for 30 seconds and displayed in kymograph format. Arrows point to a example of both G3BP1 and mRNA co-trafficking with lysosomes. Scale bar: 5 μm. (B) Kymographs showing the effect of ALS-associated ANXA11 mutants on RNA co-trafficking with lysosomes in rat neuron axons. Rat cortical neurons were transduced with LAMP1-HaloTag to label lysosomes (red), actin-24xMBS/ MCP-NLS-2xEGFP (green) and ANXA11 (upper panel) or ANXA11 (D40G) (bottom panel). Time-lapse movies of axons were then acquired at 100ms/frame for 30 seconds and displayed in kymograph format. Arrows point to an example of RNA granule co-trafficking with lysosomes. Scale bar: 5 μm. Quantification of RNA granule co-localization or co-trafficking with lysosomes in axons expressing either ANXA11 or ANXA11 p. D40G mutation (relative to lysosome number). n=36 (WT), 12 (D40G). One-way ANOVA. ns, not significant. Error bars = SEM. (C) Quantification of growth cone area in axons expressing either ANXA11 or mutant ANXA11. N=61(WT), 56(D40G), 46(R235Q), 39(R346C). One-way ANOVA. ^∗∗^, p<0.01, ns, not significant. Error bars = SEM. (D) Quantification of growth cone area in axons expressing either ANXA11 or mutant ANXA11. N= 80(sh control), 54(sh ANXA11). unpaired t-test. ns, not significant. Error bars = SEM. (E) Quantification of vesicles triple labeled with CAPRIN1/LAMP1/ANXA11 over the total LAMP1 vesicles undergoing anterograde or retrograde transport in zebrafish axons. N=9-18. (F) Quantification of trafficking distance for CAPRIN1 vesicles in zebrafish axons. N=9-18. Two-way ANOVA with Tukey post-hoc analysis, ns, not significant. ^∗^p < 0.05. Error bars = SEM. (G) Quantification of trafficking velocity for CAPRIN1 vesicles in zebrafish axons. N=9-18. Two-way ANOVA with Tukey post-hoc analysis. ^∗^p < 0.05. Error bars = SEM.

We used live-cell microscopy to test whether ANXA11 mutations altered RNA granule hitchhiking on lysosomes. In control neurons, ANXA11 co-trafficked with both lysosomes and RNA granule markers (Video S4). As predicted, the C-terminal ALS-associated ANXA11 mutation p.R235Q drastically reduced the number of trafficking RNA granules on lysosomes in axons (Figures 7A and 7B and Figure S7B, Video S5). Importantly, mutant ANXA11 expression did not alter axonal lysosome transport itself (Figure S5C). Similar to our observations in ANXA11 knockdown neurons, we found that ANXA11 mutations reduced levels of actin mRNA in growth cones per smFISH imaging (Figures 7C and 7D). These data indicate that ALS-associated mutations impair axonal RNA granule transport, as well as delivery of mRNAs to distal regions of the cell.

Video S5. ALS-associated ANXA11 mutations disrupt RNA granule/lysosome co-trafficking in primary rat neuron axons. Related to Figure 7

To determine whether ANXA11 mutations altered RNA granule axonal trafficking *in vivo*, we co-imaged lysosomes, ANXA11 and CAPRIN1 (an axonal RNA granule protein) in axons of live zebrafish neurons (Figure 7E). Both anterograde- and retrograde-moving RNA granules co-trafficked with lysosomes (Figure 7F, Video S6), and numerous motile RNA granules also co-localized with detectable levels of ANXA11 (Figure 7G, Video S7). Similar to observations in primary rat neurons, ALS-associated ANXA11 mutations interfered with RNA granule motility (Figure 7H, Figures S7E and S7G). Therefore, in primary rat neurons and in an *in vivo* zebrafish model, ANXA11 co-localizes with RNA granules and lysosomes during axonal transport. Moreover, ALS-associated ANXA11 mutations disrupt this trafficking by preventing RNA granule hitchhiking on lysosomes.

Video S6. Caprin co-trafficking with lysosomes in zebrafish axons. Related to Figure 7

Video S7. Caprin co-trafficking with ANXA11 in zebrafish axons. Related to Figure 7

## Discussion

Polarized cells such as neurons rely on active, microtubule-directed RNA transport to facilitate local protein translation at subcellular locations far from the nucleus. Here, we discovered that membraneless RNA granules hitchhike on moving lysosomes during long-distance transport in both non-polarized cells and neurons, consistent with similar recent observations by others (Gershoni-Emek et al., 2018, Cioni et al., 2019). We then identified ANXA11 as a molecular tether that couples RNA granules to lysosomes, thus mediating efficient long-distance transport of RNA. Finally, we showed that ALS-associated ANXA11 mutations alter ANXA11’s biophysical and cellular properties, impeding axonal RNA transport. Based on our observations, we propose a mechanism governing active RNA granule transport in which lysosomes recruit RNA granules through the tethering function of ANXA11, facilitating RNA granule transport to distal reaches of the cell.

For simplicity, we referred to all LAMP1-positive vesicles as lysosomes, though recent evidence suggests that LAMP1-positive structures in neurons encompass a heterogenous group of proteolytically-active and inactive vesicles (Cheng et al., 2018, Farías et al., 2017). We observed that only a fraction of LAMP1-positive vesicles co-trafficked with RNA granules, and it will be important to further characterize the precise nature of these LAMP1-positive organelles. For example, as anterograde-directed RNA granules presumably fuel local translation at distal sites, it is possible that their associated LAMP1-positive organelles are pH-neutral and non-degradative in function. By contrast, LAMP1-positive organelles near the soma have a lower pH and can either fuse with or mature into lysosomes with degradative activity (Farías et al., 2017). Our CLEM imaging showed that the bulk of RNA granules are not internalized within lysosomes, as would be expected if they had undergone bulk autophagy. However, the juxtaposition of lysosomes with RNA granules could provide an opportunity for localized internalization and degradation of portions of RNA granules (e.g. via piece-meal microautophagy). Alternatively, retrograde-directed RNA granules may function in a recycling capacity, in which no RNA granule internalization by LAMP1-positive organelles would occur.

Structural modeling of ANXA11 revealed several unique attributes suitable for its role in tethering RNA granules with lysosomes, and we found that these structural properties governed ANXA11’s overall function. Similar to many other RNA granule proteins, the N terminus of ANXA11 has a prion-like low complexity domain. Such domains can facilitate context-dependent multimerization, which in turn triggers phase separation into liquid droplets and hydrogels. However, unlike any other RNA granule-associated proteins described to date, ANXA11 also has a series of Ca^2+^ and lipid-binding, C-terminal annexin repeats. This unique combination of a low-complexity domain and a Ca^2+^-dependent membrane binding domain allows ANXA11 to interact with both RNA granules and lysosomes. Interestingly, it was recently discovered that Ca^2+^-dependent clustering of synaptic vesicles is driven by phase separation of synapsin 1, a protein that, like ANXA11, can both bind membranes and undergo phase transitions (Milovanovic et al. 2018). It is possible that additional adapter proteins regulate interactions between other membraneless and membrane-bound organelles in similar ways.

We observed that ANXA11 interacted with lysosomes only in the presence of both Ca^2+^ and the membrane phosphoinositide PI(3,5)P_2_. Interestingly, PI(3,5)P_2_ is enriched in late endosomes and lysosomes (Ikonomov et al., 2009, Michell et al., 2006), and is a known natural agonist of TRPML1, a major lysosomal calcium channel (Dong et al., 2010). Therefore, ANXA11 may bind to lysosomal membranes in response to the highly-regulated focal release of Ca^2+^ from TRPML channels. Such a mechanism could enable precise spatiotemporal recruitment and/or release of RNA granules. Indeed, we showed that a TRPML agonist can cause increased recruitment of ANXA11 to lysosomes. One possibility, therefore, is that RNA granule loading occurs at times and places of high TRPML1 activity, while RNA granule unloading occurs at times and places of TRPML1 inactivation. Molecular mediators of PI(3,5)P_2_ dynamics, such as the phosphatidylinositol-5-kinase PIKfyve, myotubularin family 3-phosphatases, and the 5-phosphatase FIG4, could also be involved in ANXA11 regulation.

Analyzing the association of ANXA11 with RNA granules, we observed that ANXA11 localizes both within the core of RNA granules and at its periphery, extending to sites in close association with lysosomal membranes. Localization of ANXA11 to the peripheral regions of these granules could facilitate interactions with lysosomes or other ANXA11-studded RNA granules during fusion events. We found that recombinant ANXA11 facilitated interactions between purified stress granule “cores” (Jain et al., 2016, Khong et al., 2017) and liposomes *in vitro*. Additionally, we observed stress-induced ANXA11 foci within cells that did not contain other markers of RNA granules. These ANXA11 foci fused with larger stress granules positive for both ANXA11 and other stress granule markers. The explanation for these unexpected distribution characteristics of ANXA11 in granules remains to be further investigated, but various possibilities can be envisioned given the previously described hierarchical organization of proteins within stress granules (Jain et al., 2016). For example, ANXA11 might participate in promiscuous interactions with low-complexity domains in other granule proteins, it might have specific interactions with local structures in low complexity domains of other granule proteins, or it might interact primarily with granule-associated RNA secondary structures (Khong and Parker, 2018, Mittag and Parker, 2018, Langdon et al., 2018, Van Treeck and Parker, 2018). Interestingly, prior global proteomic mapping of RNA binding proteins in cancer cells identified ANXA11 as an RNA binding protein (Baltz et al., 2012). It remains unclear whether ANXA11 directly binds RNA, or interacts indirectly through intercalation into RNA granules.

Mutations in ANXA11 cause ALS and a related neurodegenerative disorder, frontotemporal dementia (FTD). Numerous mutations in ANXA11 have now been described by several different groups, and may account for up to 6% of familial ALS in Chinese populations (Smith et al., 2017, Tsai et al., 2018, Zhang et al., 2018a). Pathogenic mutations occur in both the N-terminal low complexity region and the C-terminal membrane binding region. We found that these mutations altered several fundamental biophysical properties of ANXA11. Both N-terminal and C-terminal mutations increased the propensity of ANXA11 to form hydrogel-like structures, and C-terminal mutations reduced the affinity of ANXA11 for phospholipid membranes.

The overall impact of these mutation-induced biophysical changes had several consequences for RNA granule/lysosome behavior within cells. These mutations interfered with RNA granule/lysosome docking and increased the gel-like properties of ANXA11 and other associated RNA granule proteins in cells. C-terminal mutations had particularly deleterious effects on ANXA11 function, potentially because they altered both its phase separation properties and lysosomal interactions. Because we saw that ANXA11 knockdown reduced axonal RNA granule transport, it is likely that both loss-of-function and gain-of-function mechanisms contribute to ALS pathogenesis in the setting of ANXA11 mutations. We also showed that ANXA11 knockdown reduced delivery of essential mRNAs to distal regions of the neuron. We postulate that the consequences of even modestly-impaired RNA transport could, over time, lead to widespread disruption of neuronal homeostasis and potential dysregulation of synaptic activity. Concurrently, ANXA11 mutation-induced aggregates might sequester additional critical RNA granule proteins, including granule chaperones. This could lead to further dysregulation of RNA metabolism within affected cells.

In summary, our study identifies a previously unrecognized relationship between lysosomal biology and RNA metabolism, and implicates dysfunctional RNA granule trafficking as a potential converging disease mechanism in ALS. Our findings further suggest the possibility of additional mechanistic relationships between other ALS-associated genes, including those regulating lysosomal homeostasis, docking and transport machinery, and/or the biophysical state of RNA granules.

## STAR★Methods

### Key Resources Table

**Table:** 

REAGENT or RESOURCE	SOURCE	IDENTIFIER
**Antibodies**		

Mouse anti-human LAMP1 monoclonal antibody	Developmental Studies Hybridoma Bank	Cat#h4a3; RRID: AB_528126
Mouse monoclonal M2 anti-Flag antibody	Millipore Sigma	Cat#F3165; RRID: AB_259529
GST Tag Monoclonal Antibody (8-326)	Invitrogen	Cat#MA4-004; RRID: AB_10979611
ANXA11 Antibody (OTI1C6), TrueMAB Mouse Monoclonal	Thermo Fisher	Cat#CF500950
Polyclonal rabbit anti-ANXA11	Sigma-Aldrich	Cat#HPA027545; RRID: AB_1844851
TDP-43 Polyclonal Antibody	Proteintech	Cat#10782-AP; RRID: AB_615042
G3BP1 Polyclonal Antibody	Proteintech	Cat#13057-2-AP; RRID: AB_2232034
Streptavidin Alexa 488	Thermo Fisher	Cat#S32354; RRID: AB_2315383

**Bacterial and Virus Strains**		

*E. coli* BL21(DE3)	NEB	C2527I

**Chemicals, Peptides, and Recombinant Proteins**		

ML-SA1	Sigma-Aldrich	SML0627
BAPTA-AM	AAT Bioquest	CAS 126150-97-8
YM-201636	Cayman	371942-69-7
Nocodazole	Sigma	M1404
Phenol-biotin	Adipogen	41994-02-9

**Critical Commercial Assays**		

PIP strips	Echelon Biosciences	P-6001

**Deposited Data**		

LAMP1-Apex Mass spectrometry-based proteomics datasets	This paper	PeptideAtlas: PASS01313
G3BP1- Apex Mass spectrometry-based proteomics datasets	Markmiller et al., 2018	MassIVE repository: MSV000081554

**Experimental Models: Cell Lines**		

WTC11 hiPSC lines with doxycycline-inducible mNGN2 transgene at the AAVS1 locus	Fernandopulle et al., 2018	N/A

**Experimental Models: Organisms/Strains**		

Rat (Sprague Dawley)	Charles River	N/A
TgBAC (neurod:egfp)^nl1^ transgenic zebrafish	Obholzer et al., 2008	N/A
AB^∗^ WT zebrafish	ZIRC	ZL1

**Oligonucleotides**		

5'-Cy3-Oligo d(T)30	Gene link	26-4330-02
Stellaris FISH Probes, Custom Assay with Quasar 570 Dye: rat ActB (Table S1)	Biosearch Technologies	SMF-1063-5
shRNA target sequence: rat ANXA11 (Table S1)	This paper	N/A

**Recombinant DNA**		

CRY2oligo-mcherry-ANXA11	This paper	N/A
CMV-LAMP1-HaloTag	This paper	N/A
CMV-mcherry-G3BP1	This paper	N/A
CMV-ANXA11-mEmerald	This paper	N/A
MCP-NLS-2^∗^EGFP	Yoon et al., 2016	N/A
EF1a-actin-MS2 reporter	Modified from Yoon et al., 2016	N/A
PLEX-PGK-ANXA11-mEmerald	This paper	N/A
PLEX-ANXA11-mCerulean3	This paper	N/A
PLEX-PGK-LAMP1-HaloTag	This paper	N/A
PLEX-PGK-mEmerald-G3BP1	This paper	N/A
PLEX-mCherry-G3BP1	This paper	N/A
PLEX-PGK-mEmerald-caprin1	This paper	N/A
PLEX-PGK-mEmerald-TDP43	This paper	N/A
PLKO.1-sh ratANXA11	This paper	N/A
PLKO-1-sh control	This paper	N/A
PLEX-PGK-ANXA11-mCerulean3	This paper	N/A
PLEX-PGK-LAMP1-YFP	This paper	N/A
pOPINS-ANXA11(WT)	This paper	N/A
pOPINS-ANXA11(D40G)	This paper	N/A
pOPINS-ANXA11(R235Q)	This paper	N/A
pOPINS-ANXA11(R346C)	This paper	N/A
pOPINS-ANXA11(LC-aa1-185)	This paper	N/A
pOPINS-ANXA11(CTF-aa186-505)	This paper	N/A
CLYBL-LAMP1-APEX2	This paper	N/A
CLYBL-NES-APEX2	This paper	N/A
CLYBL-L-talen	addgene	pZT-C13-L1
CLYBL-R-talen	addgene	pZT-C13-R1

**Software and Algorithms**		

(Fiji is just) ImageJ 2.0.0	NIH	https://imagej.nih.gov/ij/
Graphpad Prism 5	Graphpad	https://www.graphpad.com/
ZEN Zeiss	Zeiss	N/A
MaxQuant1.6.2.3	Tyanova et al., 2016	N/A
ToppGene Suite	Chen et al., 2009	N/A
MSstats	Choi et al., 2014	N/A

**Other**		

NbActiv4 minus Phenol	Brainbits	NB4-pr
Poly-L-ornithine	SigmaAldrich	P3655
BrainPhys Without Phenol Red	Stemcell technologies	05791
ROCK inhibitor Y-27632	Selleckchem	S1049
Essential 8 medium	Thermo Fisher Scientific	A1517001
Growth factor reduced Matrigel	BD Bioscience	35881
DMEM/ F12	Thermo Fisher Scientific	11320033
N-2 supplement	Thermo Fisher Scientific	17502001
NEAA (nonessential amino acids)	Thermo Fisher Scientific	11140076
Gluta-MAX supplement	Thermo Fisher Scientific	35050061
B-27	Thermo Fisher Scientific	17504044
Recombinant Human BDNF	PeproTech	450-02
Recombinant Human NT-3	PeproTech	450-03
Laminin Mouse Protein	Thermo Fischer Scientific	23017-015

### Lead Contact and Materials Availability

Further information and requests for resources and reagents should be directed to the Lead Contact, Michael E. Ward (wardme@nih.gov). Plasmids generated in this study have been deposited to Addgene. iPSC lines will be distributed to interested parties upon request.

### Experimental Model and Subject Details

#### hiPSC culture

The control male WTC11 human induced pluripotent stem cells (hiPSC) line was obtained from Coriell. We adhered to NIH Intramural Research Program policies regarding the registration and use of this iPSC line. Karyotyping was used to authenticate that the line had a normal male karyotype. HiPSCs were maintained under feeder-free conditions in a 37^o^C, 5% CO2 tissue culture incubator on tissue culture treated dishes coated with growth factor-reduced Matrigel (BD Biosciences) and fed every 1-2 days with Essential 8 medium (Thermo Fisher Scientific), as needed. Accutase (STEMCELL Technologies) was used to enzymatically dissociate hiPSCs into single cells, and 0.5mM EDTA was used for routine dissociation to maintain colony growth. To promote cell survival during passaging, cells were passaged with the p160-Rho-associated coiled coil kinase (ROCK) inhibitor Y-27632 (10 μM: Selleckchem). hiPSCs were frozen in 90% fetal bovine serum (HyClone) and 10% DMSO (Sigma).

#### Generation of Stable hiPSC Lines

WTC11 hiPSCs with single-copy integration of a doxycycline-inducible NGN2 cassette at the AAVS1 locus (Fernandopulle et al, 2018) were singularized with Accutase, resuspended in PBS, and counted with a Countess automatic cell counter (Life Technologies). For plasmid transfections, 1.5 million hiPSCs were seeded onto one well of a 6-well dish in Essential 8 supplemented with Y-27632 (10 μM). 2-5 hours later, Lipofectamine Stem (Thermo Fisher Scientific) was used to introduce the appropriate knockin vector (CLYBL-LAMP1-APEX2 or CLYBL-LAMP1-NES-APEX2) (1.8 μg) and each CLYBL TALEN pair (0.6 μg each). Cells were dissociated the next day onto a 10cm dish and maintained for 1 week in Essential 8 medium (supplemented with 10 μM Y-27632 for the first 2 days). Single fluorescent cells were then isolated and seeded in individual wells of a 96-well dish by FACS on a Sony SH800S Cell Sorter. These cells were maintained in Essential 8 Flex medium (Thermo Fisher Scientific) supplemented with RevitaCell (Thermo Fisher Scientific). After 1-2 weeks, clones were dissociated with Accutase and seeded into 6-well dishes in Essential 8 medium supplemented with Y-27632. Genomic DNA was isolated with a Quick-DNA Microprep Kit (Zymo Research) and single-copy CLYBL integration was confirmed with PCR. PCR positive clones were subsequently checked for genomic abnormalities with karyotyping.

#### Inducible System for Neuron Differentiation

Stable hiPSC lines was generated from the WTC11 genetic background with a doxycycline-inducible mNGN2 transgene at the AAVS1 locus. For differentiation, 25 million hiPSCs were seeded onto a 15cm tissue culture dish in Neuronal Induction Medium (NIM), composed of DMEM/F12 medium (ThermoFisher Scientific), N-2 supplement (ThermoFisher Scientific), Nonessential amino acids supplement (NEAA) (ThermoFisher Scientific), Gluta-MAX supplement (ThermoFisher Scientific), Y-27632 (10 μM), and doxycycline (2 μg/ml, Sigma). Cells were maintained on NIM with daily full medium changes for 3 days.

#### hiPSC-derived Neuron Culture

After the 3-day differentiation period, cells were dissociated with Accutase from the 15-cm dish and seeded onto final experimental plates coated with poly-L-ornithine (0.1 mg/ml). Cells were seeded and maintained in Cortical Neuron Culture Media, composed of BrainPhys Neuronal Medium (STEMCELL Technologies), B-27 supplement (ThermoFisher Scientific), brain-derived neurotrophic factor (10 ng/ml), neurotrophin-3 (10 ng/ml), and mouse laminin (1 μg/ml). Half-media changes were conducted every 3 days for the lifetime of the culture.

#### U2OS cell culture

The U2OS cell line used in these studies was the human osteosarcoma cell line, obtained directly from ATCC (HTB96). Cells were authenticated by morphological assessment under microscopy. Cells were grown in a 37^o^C, 5% CO2 tissue culture incubator on tissue culture treated dishes in DMEM + 10% FCS and passaged with Trypsin EDTA.

#### Primary Cortical Neuron Culture

Cortices were dissected from E17 Sprague-Dawley rat embryos of both sexes. Rat maintenance and care followed policies advocated by NRC and PHS publications, and approved by Institutional Animal Care and Use Committee (IACUC), Janelia Research Campus.

Tissue were digested with papain and gently triturated and filtered through 70 micron strainer. Neurons were plated in poly-L-ornithine coated dishes and cultured in Nbactive4 medium at 37^o^ C, indicated virus were transduced at DIV 10-14 and imaged at DIV 17-21.

#### Zebrafish husbandry

Adult ^∗^AB were maintained at 28.5^o^C and spawned according to standard protocols (Kimmel et al., 1995). Embryos, 3 days post-fertilization (dpf), were derived from natural matings or *in vitro* fertilization, raised in embryo media, and developmentally staged using previously established methods (Westerfield, 2000). All *in vivo* experimental protocols were approved by the National Institute of Child Health and Human Development Animal Care and Use Committee (ASP18.008).

### Method Details

#### Plasmids and Cloning

All plasmids generated in this study have been deposited to Addgene. Plasmids for generating stable iPSC lines (e.g. APEX2 constructs) were designed with 1 kb left and right homology arms against the CLYBL (citrate lyase subunit beta-like protein, Uniprot ID: Q8N0X4) locus. This locus was chosen to permit stable expression in both the iPSC and neuron stages. Plasmids were generated with PCR and recombinase-based cloning (In-Fusion by Clontech or NEBuilder by NEB). Plasmids for virus transduction in neurons (e.g. ANXA11-mEmerald, ANXA11-mCerulean3 LAMP1-HaloTag, LAMP1-YFP, mcherry-G3bp1,mEmerald-G3bp1, mcherry-TDP43, mEmerald-TDP43, mEmerald-caprin1) were generated with the pLEX lentiviral vector (Thermo Scientific) and ligation-based cloning. Actin-24xMBS, MCP-NLS-HaloTag and MCP-NLS-2xEGFP plasmids were kindly provided by Dr. Young Yoon from Dr. Robert H. Singer lab. Opto-ANXA11 plasmids were made by insertion of CRY2olig-mcherry on the N-terminal of ANXA11 plasmids by Gibson cloning. ANXA11 shRNA expression cassettes were made by oligo annealing and ligation into pLKO.1 vector.

#### Antibodies

Mouse monoclonal anti-Lamp1 antibody (H4A3, Developmental Studies Hybridoma Bank) was used extensively for western blot (WB) and immunofluorescence (IF) studies, and mouse monoclonal M2 anti-Flag antibody (F3165, Millipore Sigma) was used to detect Lamp1-APEX expression via IF. GST Tag monoclonal antibody (8-326, ThermoFisher) was used to detect ANXA11-GST in lipid strip assay. Polyclonal rabbit anti-ANXA11 (HPA027545) was used for WB in lysosomal isolation experiments, liposome floatation as well as for protein localization via IF. Polyclonal rabbit anti-TDP43 (10782-AP, Proteintech) and anti-G3BP1 (13057-2-AP, Proteintech) were used to detect stress granule formation via IF, and anti-G3BP1 was also used for stress granule isolation (IP) and WB.

#### APEX proteomics

hiPSC-derived neurons were grown to a density of 10 million cells per 10cm dish. 4 biological replicates of each experimental condition were used. To provide a substrate for the APEX enzyme, cells were fed with 500uM phenol-biotin (CAS 41994-02-9, Adipogen) and incubated at 37C for 30 minutes prior to enzyme stimulation. To stimulate APEX2, cells were treated with 1mM hydrogen peroxide and incubated at 37C for 1 minute. The reaction was terminated by aspirating the growth medium and adding 4mL of ice-cold quench buffer (10mM sodium azide, 10mM sodium ascorbate, 5mM TROLOX in PBS) 3 times before lysing the cells with 600uL of Lysis buffer (50mM Tris-Cl pH 7.4, 500mM NaCl, 0.2% SDS, 1mM DTT, 10mM sodium azide, 10mM sodium ascorbate, 5mM TROLOX, cOmplete mini protease inhibitor tablets, in MS-grade H_2_O). Cells were transferred to 4C room and incubated on a nutator until all stimulations were finished. Cells were scraped into 1.5mL polystyrene microcentrifuge tubes and sonicated (QSonica Q800R2) for 14 minutes with alternating 1-minute on/30 seconds off cycles. Lysate was spun at 4C at max speed for 12 minutes, and the supernatant was diluted with one volume of 50mM Tris-HCl. Samples were dialyzed to remove excess biotin and detergents. Dialysis buffer composed as follows: 50mM Tris-Cl pH 7.4, 250mM NaCl, 0.1% SDS, 1% Triton X-100 in H2O. Samples were loaded into SnakeSkin dialysis tubing (Thermo Fisher Scientific) and placed within a 2L dialysis chamber with a spin bar at 4C. Samples were dialyzed at 4C for 4 hours, with dialysis solution replaced every hour. After dialysis, the protein concentration was quantified using a Bio-Rad DCA assay, and a bead titration was carried out to determine the optimal volume of beads to use for complete pulldown of biotinylated proteins. We used Streptavidin Sepharose High Performance (GE Healthcare Life Sciences) beads for Lamp1-APEX experiments, and Nanolink Streptavidin Magnetic beads (Solulink) for ANXA11-APEX experiments. After normalization of protein amount and determination of optimal bead volume for each sample, bead incubation occurred overnight with agitation at 4C. The next day, beads were washed with 4 sequential buffers (Buffer 1: 2% SDS; Buffer 2: 50mM Tris-HCl pH 7.4, 00mM NaCl, 0.1% deoxycholic acid, 1% Triton-X, 1mM EDTA; Buffer 3: 10mM Tris-HCl pH 7.4, 250mM NaCl, 0.5% deoxycholic acid, 0.5% NP-40, 1mM EDTA; Buffer 4: 50mM Ammonium bicarbonate; *all in ddH*_*2*_*O*). Then, proteins were reduced (5mM TCEP, 30 minutes), alkylated (15mM iodoacetamide, 30 min), quenched (5mM DTT, 15 min), and digested on beads overnight for 14 hrs with 1ug of trypsin/LysC enzyme (Promega). The next morning, 0.5ug of trypsin/LysC was added and incubated for another 2 hours at 37C at 1200 rpm. Samples were separated from beads with either centrifugation or magnetic separation, the supernatant was acidified with 10% trifluoroacetic acid (TFA), and samples were desalted using C18 solid-phase extraction spin columns. After eluting samples in 50% acetonitrile (ACN)/0.1% TFA, they were dried down in a speedVac and then resuspended in 2% ACN/0.1% formic acid (FA) for subsequent LC-MS analysis.

#### LC-MS analysis

Peptide samples were analyzed on a Dionex UltiMate 3000 RSLCnano system coupled with a Thermo Scientific Q-Exactive HF mass spectrometer. Mobile phase A was 0.1% FA, 5% DMSO in H_2_O, and mobile phase B was 0.1% FA, 5% DMSO in ACN. Flow rate was 0.3 μL/min. Peptide separation was achieved on a PepMap C18 column (2 μM, 100Å, 75μM×25 cm) with a 120 min LC gradient. Top 15 data-dependent acquisition (DDA) was conducted, and the MS was scanned from *m/z* 350 to1500 at a resolving power (RP) of 120K. Parent masses were isolated (*m/z* 1.4 window) and fragmented with higher-energy collisional dissociation (HCD) with a normalized collision energy (NCE) of 27%. Dynamic exclusion time was 22.5 s. Automatic gain control (AGC) targets were 1 × 10^6^ for MS and 2 × 10^5^ for MS/MS acquisitions. Maximum injection times (maxIT) were 30 ms for MS and 35 ms for MS/MS.

#### ANXA11 homology models

The full-length sequence of human ANXA11 (SWISSPROT code P50995) was submitted to the iTasser web server on 05/23/18 to identify suitable templates for structural modeling. The predicted secondary structure and unfolded nature of the resultant structural models confirmed the assignment of the N-terminal domain (residues 1-199) as unstructured. Template structures with good coverage of the C-terminal segment were all annexins, of which the candidate with the highest similarity was human annexin A4 (∼57% identical residues). Among the available structures of ANXA4, PDB code 2ZOC has the highest resolution (2.0 Å) and contains four resolved calcium ions. To construct models of ANXA11 C-terminal domain with and without bound calcium ions, we used the pairwise sequence alignment obtained from iTasser (which is gapless for residues 5-319 of 2ZOC to residues 192-505 of ANXA11). Models were built using Modeller 9v19, with and without calcium ions in the same positions as in the template. Distance restraints were included in Modeller to optimize the calcium-oxygen interaction distances in the calcium binding sites, using upper bounds extracted from the equivalent interaction distances in the template. In each case, 100 models were built and ranked according to their MolPDF scores, which provides an assessment of the extent to which the model satisfies the restraints. We filtered out models with MolPDF scores outside the standard deviation obtained over all 100 models (excluding 9 and 14 models for the apo and holo, respectively). The remaining models were filtered to consider only the 20% models with the highest ProQ2 scores (> 0.928). The ProQ2 score of the template is 1.005, for reference. Finally, we identified two models with the fewest backbone dihedral angles in the disallowed region of the Ramachandran plot, using PROCHECK. The holo and apo model have 96.9% and 96.5% residues, respectively, in the allowed regions of the Ramachandran plot, compared to 93.8% for the template.

#### Continuum electrostatics calculations

Electrostatic potential surfaces were computed as follows. Partial charges and atomic radii were obtained for the model coordinate files using the PDB2PQR server v2.1.1, with the CHARMM force field, which includes calcium parameters. Poisson-Boltzmann electrostatic potentials were computed using the APBS plugin within Pymol using default parameters.

#### Expression and purification of ANXA11

Constructs encoding ANXA11 residues 1-505 and its mutants (D40G, R235Q, R346C), Annexin LC (aa1-185) and CTF (aa186-505), were either cloned into pOPINS vector containing an N-terminal His-Sumo Tag and a ULP protease cleavage site or in pACEBac2 vector with a TEV cleavable N-terminal MBP tag and an mCherry-6xHis-C-terminal tag. ANXA11 (R235) mutant protein was highly aggregation prone and could not be purified, so no further *in vitro* studies were conducted with this mutant. His-Sumo tagged proteins were expressed in *E. coli* BL21(DE3) in TB autoinduction media by an overnight incubation at 25°C. Briefly, cells were centrifuged and lysed using a high pressure cell disruption system. Clarified lysate was loaded onto a Ni-Sepharose Excel column and purified using standard procedure. Protein containing eluates were pooled, and dialysed in 50mM HEPES pH 7.4, 100mM NaCl, and 5% glycerol buffer after addition of ULP protease to remove the His-Sumo Tag. Protein was further purified on a second Ni-sepharose column to remove the His-Sumo tag followed by a size-exclusion column and the fractions containing purified protein were pooled for all subsequent experiments. mCherry tagged Annexin WT and its mutants constructs were expressed and purified from insect Sf9 cells using standard procedures. After 6 days of infection cells were harvested and lysed by homogenising into a resuspension buffer containing 50mM HEPES pH 7.4, 100mM NaCl, and 5% glycerol, 0.1% CHAPS. Cell Lysates were subjected to high speed centrifugation and the clarified lysate was subsequently purified using three steps purification protocol including, Ni- Sepharose Excel affinity resin, Amylose resin, followed by size exclusion chromatography in the buffer containing 50 mM HEPES, 225mM NaCl pH 7.4.

#### ANXA11 droplet assay

Phase separation of ANXA11 WT and it mutants was initiated either by changing the temperature of the samples from 4°C to RT or by the addition of the crowding reagent,10% dextran. Purified ANXA11 protein concentration ranging from 0.1 μM-50 μM in a total volume of 20 μL were deposited on 8-well glass bottom Ibidi slides, incubated at room temperature for ≥30 minutes before being imaged on a Zeiss Axiovert 200M microscope with Improvision Openlab software using 100X magnification objective. ImageJ software was used in all image processing. For all purified proteins n ≥ 3.

#### In vitro liposome RNA granule reconstitution assay

Stress granule cores were purified according to a previously-published method (Jain et al., 2016, Khong et al., 2017, Wheeler et al., 2017) from SH-SY5Y cells expressing Emerald-G3BP1 after 1h heat shock at 43^o^C. Stress granule cores were incubated with liposomes in the presence of 1 μM ANXA11 only, 500 μM calcium only, or both 1 μM ANXA11 and 500 μM calcium at 37^o^C for 1 hour.

#### Liposome preparation

Liposomes were prepared with commercially available lipid components from Avanti Polar Lipids. Liposome composition was 70% phosphatidylcholine (Avanti #850457C), 24% phosphatidylethanolamine (Avanti #850757C), 5% cholesterol (Avanti #700000), and 1% phosphatidyl-3',5'-bisphosphate (Avanti 850164P). Powdered lipids were individually resuspended in chloroform, then mixed together in a glass sample vial and evaporated with a dry N_2_ stream. The mixture was resuspended in a buffered salt solution (10mM Na-HEPES, 50mM NaCl, 1mM EDTA, pH 7.4) for 30 minutes, and then sonicated in a water bath for 3 minutes to complete resuspension. The lipid mixtures were then extruded into large unilamellar vesicles (LUVs) using an Avanti Mini-Extruder (Avanti #610023) that was heated to 60^o^C. For the liposome preparation for microfluidic assay, The composition of liposomes without PIP is POPC: 49%, POPS: 10%, POPE: 20%, SAPI: 15%, Cholesterol: 5% DOPE-ATTO647: 1%. The composition of liposomes with PIP3 is POPC: 49%, POPS: 10%, POPE: 20%, SAPI: 10%, PIP 5%, Cholesterol: 5% DOPE-ATTO647: 1%.

Giant unilamellar vesicles were prepared from 50% phosphocholine, 10% phospho-L-serine: 20% phosphoethanolamine, 10% phosphoinositol (Avanti #850144), 5% cholesterol, 5% phosphatidylinositol 3-phosphate(PI3P, Avanti #850150), 0.05% ATTO488 labeled 1,2-dioleoyl-sn-glycero-3-phosphoethanolamine- dissolved in chloroform using electroformation method in a Nanion Technologies Ves Prep pro setup (Nanion Technologies, Munich, Germany). The lipids were purchased from Avanti (Avanti Polar Lipids, Alabaster, USA).

#### Liposome Flotation Assay

In a thick-wall polycarbonate tube, LUVs and recombinant annexin A11 protein were mixed to a final concentration of 0.5mM LUVs and 0.25uM protein in 100μL of buffered salt solution (see above). LUV-protein mixture (with or without 100μM CaCl_2_ added) was incubated for 1 hour at 4^o^C. Then, 100μL of 60% sucrose in PBS was added and mixed gently to yield a 30% final sucrose concentration. 250μL of 25% sucrose was overlaid atop the 30% sucrose layer, and then 50μL of PBS was added as the final layer in the sucrose gradient. The gradient was centrifuged at 174,000 x g at 4^o^C for 1 hour, and then the fractions (top 100μL, middle 200μL, and bottom 200μL) were separated. Sample buffer was added to each layer and resolved by electrophoresis.

#### Lipid strip assay

Membrane lipid binding analysis of GST tagged ANXA11 was conducted using membrane lipid Strips (Echelon Bioscience), with each spot containing 100 pmol of indicated lipids. Membrane were blocked by PBST with 3% BSA for 1hr at room temperature, followed by incubated with 0.25μg/ml ANXA11-GST (LSBio) for 1hrs in blocking buffer, 50μM CaCl_2_ or 1mM EGTA were added to the incubation solution as indicated. After washed 3 times with PBST, membrane was blotted with anti-GST antibody (ThermoFisher).

#### Microfluidic setup and diffusion sizing assay

The microfluidics setups used here have been described previously(Duffy et al., 1998, Gang et al., 2018, Qin et al., 2010). For these studies, microfluidic devices were fabricated by using polydimethylsiloxane (PDMS) (Sylgard 184 kit, Dow Corning, Midland, MI, U.S.A) mixed with black carbon powder (Sigma-Aldrich, Poole, U.K.) to maximize the fluorescent signal. The channel is typically with 300 μm in width and 50 μm in height. The devices were plasma-treated and modified with Tween 20 2% in ethanol solution prior to the measurements. The channels were flushed and prefilled with buffer. We loaded the sample and buffer at the inlets and withdraw liquid from the outlet using a glass syringe (Hamilton, Reno, NE, USA) mounted to a syringe pump (Cetoni neMESYS, Cetoni GmbH, Korbussen, Germany). 5 μM mCherry ANXA11 was first mixed with liposomes with 500 μM phosphate lipids at different concentrations of calcium chloride and then co-flown with the buffer along the channel. The fluorescent signal was collected at 4 points along the channel with different diffusion time in order to calculate the size of ANXA11. The images were obtained from an inverted microscope (Axio Observer A1, Zeiss, Cambridge, U.K.) coupled to a CCD camera (Evolve 512, Photometrics, Tucson, AZ, USA). The hydrodynamic radii of ANXA11 binding to liposomes were measured at different calcium concentrations with a microfluidic diffusional sizing assay. Enhanced binding was detected from 50 to 1000 μM calcium concentration for liposomes with PI3P and from 100 to 5000 μM for those without PI3P. In independent experiments, the radius of ANXA11 were determined to be equal to 5.45 ± 0.3 nm. During the short duration of diffusion experiments there was no observed influence of calcium ions on protein size, the radius of ANXA11 was 5.51 ± 0.6 nm in the presence of 500 μM CaCl_2_ and 5.27 ± 0.5 nm in the presence of 13.75 mM CaCl_2_. The radius of lipid vesicles was measured to be 14.7 ± 1.1 nm and did not significantly change in the presence of calcium concentrations used in the binding experiments.

#### Poly A RNA in situ hybridization, single molecule FISH (smFISH) and immunofluorescence staining

In brief, cells were fixed with 4% paraformaldehyde for 10 mins, then 100% cold methanol for 10 mins and followed by 70% ethanol for 10 mins for rehydration, cells were then incubated with 1M Tris pH8.0 for 5 mins before incubated with 1ng/μl Cy3-Oligo-dT (30) for rat beta-actin in hybridization buffer (2xSSC with 1mg/mL Yeast tRNA, 0.005% BSA, 10% Dextran sulfate, 25% formamide) in 37^o^C overnight. For single molecule FISH, rat ActB-Quasar570 smFISH probes were designed using the Stellaris Probe Designer software (Biosearch Technologies), smFISH was performed according to manufacturer’s instruction using Stellaris buffers (Biosearch Technologies SMF-HB1-10, SMF-WA1-60 and SMF-WB1-20). After hybridization, cells were washed with 4xSSC and 2xSSC once each, and incubated with primary antibody in 2xSSC+ 0.1% triton-X-100 at 4^o^C overnight, then washed 3 times with 2xSSC and incubated with fluorescence labeled secondary antibody in 2xSSC+ 0.1% triton-X-100 at room temperature for 1hr. Coverslips were mounted and imaged with Zeiss Airyscan.

#### Live cell imaging

Live cell imaging was carried out in phenol red free normal culture medium. For U2OS cells, imaging was performed using Zeiss 880 LSM with Airyscan, plan-apochromatic 63x oil objective (NA=1.4), images were processed with Airyscan processing in ZEN software (Zeiss). Axonal trafficking imaging was performed using Nikon spinning disk equipped with 100x oil objective lens (NA=1.4). Time-lapse movies of axons were then acquired at 100ms/frame for 30 seconds and displayed in kymograph format.

#### EM imaging of LAMP1-APEX2

i^3^Neurons stably expressing LAMP1-APEX2 were fixed with 2% glutaraldehyde (Electron Microscopy Services) in EM buffer (0.1 N sodium cacodylate at pH 7.4 with 2 mM calcium chloride) for 30 minutes. Cells were washed 3X with EM buffer and then exposed to ImmPACT DAB solution (Vector Labs) for 10 minutes. Samples were washed with EM buffer an additional 3X and then fixed with 2% glutaraldehyde for at least an additional 48 hrs. Samples were washed with buffer and treated with 1% reduced osmium tetroxide in 0.1 N cacodylate buffer at pH 7.4 for 1 h on ice, washed and *en bloc* stained with 0.25–1% uranyl acetate in 0.1 N acetate buffer at pH 5.0 overnight at 4°C, dehydrated with a series of graded ethanol and finally embedded in epoxy resins. Ultrathin sections (70 nm) were stained with lead citrate and imaged with a JEOL 1200 EXII Transmission Electron Microscope.

#### Correlative Light-Electron Microscopy

U2OS cells were plated on glass gridded coverslips (Electron Microscopy Sciences, Hatfield, PA) and transfected with indicated plasmids. 24hrs after transfection, cells were fixed in 2% glutaraldehyde, 2 mM CaCl2 in 0.08 M sodium cacodylate buffer, pH 7.2 at RT for 10 minutes and imaged on Zeiss Airyscan to collect light microscopy images. Cells were kept in fixative at 4^o^C for 16hrs and postfixed in 2% osmium tetroxide-1.25% potassium ferrocyanide in cacodylate buffer for 30 min followed by 2% osmium in cacodylate buffer for another 30 min and processed for Epon embedding. Cells imaged by Airyscan were localized on the grid (imprinted in the Epon block). Ultrathin sections (60 nm) from the imaged cells were cut and post-stained with uranyl acetate/lead citrate and imaged in a Tecnai 12 electron microscope (FEI, Hillsboro, OR) operating at 80kV equipped with an Ultrascan 4000 digital camera (Gatan Inc, CA).

#### Zebrafish Axonal Imaging

Zebrafish axonal transport analyses were done as previously described(Drerup and Nechiporuk, 2016, Mandal et al., 2018). Briefly, zygotes were injected with plasmid DNA encoding fluorescently tagged cargos of interest with expression driven by the *5kbneurod* promoter (Mandal et al., 2018). At 3 days post-fertilization (dpf) embryos were sorted under epifluorescence to identify individuals with tagged cargo expression in a few cells of the pLL ganglion. For imaging, embryos were mounted in 1.5% low melting point agarose on a glass coverslip, submerged in embryo media containing 0.02% tricaine and imaged using a 63X/NA=1.2 water objective on an upright LSM800 confocal microscope (Zeiss). A region of interest (30-200 μm) for each embryo was selected in the pLL nerve in which a long stretch of axon was observable in a single plane. Scans were taken at 3 frames per second for 500 to 1000 frames.

### Quantification and Statistical Analysis

#### Imaging Analysis

For line scan analysis in U2OS cells (Figure 3E, 3I, 4G, 6B, supplemental 3D, 3E, 3F, 6H), straight line across the RNA granule were drawn as ROI, intensity of ROI from each channel were calculated by plot profile tool in Fiji. Fields of view for imaging were randomly chosen. For FRAP analysis in U2OS cells (Figure 3C, 4B, supplemental 4I), three images were acquired prior to photobleaching followed by imaging over the course of recovery. Relative intensity of the photobleached ROI were calculated by substracting mean intensity of the background from the photobleached ROI region, followed by normalized to the mean intensity of the pre-bleach ROI, n represented number of ROI. For co-localization analysis (Figure 3E, 3I, 4H, 5B, 5D, supplemental 1G, 3E, 3F, 4E, 5B, 6B, 6C, 6G), RNA granule or organelles signals were segmented using trainable weka segmentation plugin in Fiji, area of colocalization were calculated by applying ’AND’ function in image calculator tool for the segmented signals, colocalization were determine by >1 pixel overlapping of segmented signal using analyze particle tool in Fiji, n represented number of cells. Image acquisition and analysis for ANXA11(FL, N-, C-)-RNA granule-lysosome colocalization studies were blinded. For trafficking analysis in U2OS (Figure1B, Figures S1C, S1D, S1E), RNA granule or organelles signals were segmented using trainable weka segmentation plugin in Fiji, trajectory of the segmented signals’ centroid were analyzed by Trackmate plugin in Fiji, n represents number of cells (Figure 1B) or number of trajectories (Figures S1C, S1D, S1E). For in vitro RNA granule liposomes reconstitution analysis (Figure6E), images were processed in Fiji using a custom macro. The liposome channel was initially gaussian blurred (sigma radius 1.5) to remove shot noise, thresholded and masked. The integrated fluorescence intensity of the G3BP1 channel within the liposome mask was calculated and normalized to the mask area, n represents number of independent experiments. For smFISH signal quantification in growthcone (Figures 6J, 7D, Figures S7C, S7D), areas of growthcone were masked, smFISH signal within masked area were segmented using trainable weka segmentation plugin in Fiji. Number of segmented smFISH signal were counted using analyze particle tool in Fiji, n represented number of growthcones. For rat axonal trafficking analysis (Figures 5F, 6H, 7B, Figures S1L, S1M, S1N, S5C, S6E, S7B), axonal images were straightened, resliced in Fiji to generate kymograph, trajectory of vesicles were analyzed by Trackmate plugin in Fiji. Co-trafficking events were defined using the cut-off of net displacement >10μm, n represents number of neurons. For zebrafish axonal imaging analysis (Figure 7H, Figures S7E, S7F, S7G), embryos expressing both constructs in a single cell were selected and imaged sequentially. Transport parameters were analyzed using kymograph analysis in the Metamorph software package (Molecular Devices), n represents number of zebrafish. Imaging statistical analysis were performed using Graphpad Prism 5. For statistical testing, t-tests and ANOVA tests were employed on samples with large size (>30); violation of the normality assumption were not taken into account because of central limit theorem. F-test was used to access the equality of variances of t-test, Bartlett test was use to access the equality of variances of ANOVA test.

#### LC-MS Data Analysis

LC-MS/MS (Figures 2D and 2E) raw files were processed by MaxQuant1.6.2.3 software (Tyanova et al. 2016) for peptide/protein identification and quantification. MS and MS/MS spectra were searched against the Uniprot human proteome database for identification with a false discovery rate cutoff level of 1%. Cysteine carbamidomethylation was searched as a fixed modification, and protein N-terminal acetylation, methionine oxidation as variable modifications. Maxquant output files were further analyzed by MS-Stats in R (Choi et al., 2014) for statistical analysis. ToppGene Suite (https://toppgene.cchmc.org was used for Go-term analysis (Chen et al., 2009).

#### Fitting of the Diffusional Sizing assay

Diffusional sizing experiments (Figure 2Q) were analyzed using custom written script based on previous study (Gang et al. 2018) with minor modifications. Concentration profiles were obtained from fluorescence images at 4 positions corresponding to 4 different diffusion times and diffusional profiles were fitted to one-Gaussian model. Hill cooperative binding model was used to fit the change in hydrodynamic radius upon ANXA11 binding to lipid vesicles and determine the binding constant (Equation 1):

(Eq. 1) $$y=a+\left(b-a\right)\frac{{\left[Ca\right]}^{n}}{K_{D}^{n}+{\left[Ca\right]}^{n}}$$

Where a is radius of unbound ANXA11, b is radius of vesicle-protein complex, [Ca] is calcium concentration in solution, K_D_ is dissociation constant (in M) and n is number of cooperative Ca binding sites. OriginPro 2016 was used to fit the experimental data.

### Data and Code Availability

The accession number for the mass spectrometry-based proteomics datasets reported in this paper is PeptideAtlas: PASS01313. (http://www.peptideatlas.org/PASS/PASS01313). Structural models of ANXA11 with and without bound calcium, and the input files to generate them, are available at DOI: https://doi.org/10.5281/zenodo.3368597. All other data are either available in the main article or in supplemental files.

## Supplemental Information

Data S1. LAMP1 Apex, related to Figure 2 and STAR Methods

Table S1. Oligonucleotides for FISH and shRNA targets, related to Figure 6, 7 and STAR Methods
