# Supplementary material for: RNA Granules Hitchhike on Lysosomes for Long-Distance Transport, Using Annexin A11 as a Molecular Tether
Source: Cell. 2019 Sep 19;179(1):147–164.e20. doi: 10.1016/j.cell.2019.08.050 (PMC6890474; doi:10.1016/j.cell.2019.08.050)
Supplement: Table S1. Oligonucleotides for FISH and shRNA targets, related to Figure 6, 7 and STAR Methods [file mmc2.pdf]

Stellaris® FISH Probes, Custom Assay with Quasar® 570 Dye: rat beta actin

#1: acgacgagcgcagcgatatc
#2: cacgatggaggggaagacgg
#3: ggtacttcagggtcaggatg
#4: gtgacaatgccgtgttcaat
#5: aaatcttctccatatcgtcc
#6: acacgcagctcattgtagaa
#7: cttttcacggttggccttag
#8: ggtctcaaacatgatctggg
#9: aacacagcctggatggctac
#10: gtacgaccagaggcatacag
#11: ctccggagtccatcacaatg
#12: tcatagatgggcacagtgtg
#13: cagacgcaggatggcatgag
#14: atcttcatgaggtagtctgt
#15: ctgtggtggtgaagctgtag
#16: taatgtcacgcacgatttcc
#17: catctcttgctcgaagtcta
#18: tcattgccgatagtgatgac
#19: tagtttcatggatgccacag
#20: acgtcacacttcatgatgga
#21: atagaggtctttacggatgt
#22: caccagacagcactgtgttg
#23: caatgcctgggtacatggtg
#24: gatcttcatggtgctaggag
#25: gctcaggaggagcaatgatc
#26: ccaatccacacagagtactt
#27: acagtgaggccaggatagag
#28: actcatcgtactcctgcttg
#29: agaagcatttgcggtgcac

shRNA target rat ANXA11

#1: ggatatccgagcagagtataa
#2: ggaagatcctgctgaagatct
#3: gccaacatgtctggaacattc
#4: ggtcctgtttgatgtctatga
